# Ligand-induced perturbation of the HIF-2α:ARNT dimer dynamics

**DOI:** 10.1371/journal.pcbi.1006021

**Published:** 2018-02-28

**Authors:** Stefano Motta, Claudia Minici, Dario Corrada, Laura Bonati, Alessandro Pandini

**Affiliations:** 1 Department of Earth and Environmental Sciences, University of Milano-Bicocca, Milan, Italy; 2 Department of Immunology, Transplantation, and Infectious Diseases, DIBIT Fondazione San Raffaele, Milan, Italy; 3 Department of Computer Science–Synthetic Biology Theme, Brunel University London, Uxbridge, United Kingdom; 4 The Thomas Young Centre for Theory and Simulation of Materials, London, United Kingdom; Koç University, TURKEY

## Abstract

Hypoxia inducible factors (HIFs) are transcription factors belonging to the basic helix−loop−helix PER-ARNT-SIM (bHLH-PAS) protein family with a role in sensing oxygen levels in the cell. Under hypoxia, the HIF-α degradation pathway is blocked and dimerization with the aryl hydrocarbon receptor nuclear translocator (ARNT) makes HIF-α transcriptionally active. Due to the common hypoxic environment of tumors, inhibition of this mechanism by destabilization of HIF-α:ARNT dimerization has been proposed as a promising therapeutic strategy. Following the discovery of a druggable cavity within the PAS-B domain of HIF-2α, research efforts have been directed to identify artificial ligands that can impair heterodimerization. Although the crystallographic structures of the HIF-2α:ARNT complex have elucidated the dimer architecture and the 0X3-inhibitor placement within the HIF-2α PAS-B, unveiling the inhibition mechanism requires investigation of how ligand-induced perturbations could dynamically propagate through the structure and affect dimerization. To this end, we compared evolutionary features, intrinsic dynamics and energetic properties of the dimerization interfaces of HIF-2α:ARNT in both the apo and holo forms. Residue conservation analysis highlighted inter-domain connecting elements that have a role in dimerization. Analysis of domain contributions to the dimerization energy demonstrated the importance of bHLH and PAS-A of both partners and of HIF-2α PAS-B domain in dimer stabilization. Among quaternary structure oscillations revealed by Molecular Dynamics simulations, the hinge-bending motion of the ARNT PAS-B domain around the flexible PAS-A/PAS-B linker supports a general model for ARNT dimerization in different heterodimers. Comparison of the HIF-2α:ARNT dynamics in the apo and 0X3-bound forms indicated a model of inhibition where the HIF-2α-PAS-B interfaces are destabilised as a result of water-bridged ligand-protein interactions and these local effects allosterically propagate to perturb the correlated motions of the domains and inter-domain communication. These findings will guide the design of improved inhibitors to contrast cell survival in tumor masses.

## Author summary

A low oxygen condition, called hypoxia, often occurs in tumor masses and generally correlates with worse prognosis. Cells in a tumor react to low oxygen levels with a metabolism modification induced by the activation of hypoxia inducible factors (HIFs) through dimerization with a partner protein and binding to a DNA target. Disrupting this protein-protein interaction could be a potential therapeutic strategy, but directly interfering with dimer formation can be troublesome because of the difficulty to design drugs that bind to protein interfaces. However, ligands that bind internal protein cavities can indirectly perturb the interfaces reducing dimers stability. Albeit protein crystallography had offered a detailed static picture of a HIF dimer bound to candidate inhibitors, it is not able to describe either the perturbation caused by binding or the molecular mechanism of dimer destabilization. Here we exploit molecular dynamics to identify the crucial interfaces in the HIF dimer stabilization and, by comparing the results obtained in the bound and unbound forms, we reveal the mechanism of ligand inhibition at atomic detail. All these findings will guide toward the design of improved dimerization inhibitors, to contrast cell survival in tumor masses.

## Introduction

Hypoxia inducible factors (HIFs) are obligate heterodimers belonging to the basic helix−loop−helix (bHLH) superfamily of transcription factors that mediate the physiological responses to hypoxia. This extensive protein family is characterized by a 4–6 basic amino acids next to a HLH dimerization domain, both required to properly bind DNA targets. Within the bHLH superfamily HIFs belong to the subfamily containing the PER/aryl hydrocarbon receptor nuclear translocator (ARNT)/single minded (SIM) (PAS) homology domain (bHLH-PAS) [1–3]. Based on their heterodimerization behavior, bHLH-PAS proteins can be further divided into two classes: class I members only form heterodimers with a member of class II, which, by contrast, can promiscuously homo- and heterodimerize. Class I includes aryl hydrocarbon receptor (AhR), aryl hydrocarbon receptor repressor (AhRR), neuronal PAS proteins (NPAS1-4), single minded proteins (SIM1-2), clock circadian regulator (CLOCK) and three HIF-α subunits isoforms, HIF-1α, HIF-2α, and HIF-3α, each targeting both shared and distinct genes [4]. When transcriptionally active, HIF-α subunit dimerizes with the constitutive ARNT (also known as HIF-β) subunit, the best characterized class II protein; other members of this class include the tissue restricted ARNT2, and the circadian rhythm proteins BMAL1 and BMAL2 [2,3].

The poorly conserved C-terminal region of bHLH-PAS proteins hosts the transactivation domains (TAD) where the transcriptional coactivators are recruited to initiate the transcription [1]; the N-terminal part contains three well-defined domains: bHLH, PAS-A, and PAS-B. The bHLH domain offers the primary dimerization interfaces and, together with the protein partner, determines the target gene recognition [5]. Despite low sequence identity, the PAS domains show conserved three-dimensional structures in a wide range of prokaryotic and eukaryotic proteins [2]. They contribute to the dimerization and increase the specificity of partner choice [6,7]. PAS-A, in particular, prevents dimerization with non- PAS-containing bHLH proteins and participate to the binding of DNA sequences that differ from the prototypical E-box motif [5]. The PAS-B domain commonly functions as a signalling domain and hosts hydrophobic cavities for small molecules and/or cofactors that relay environmental or metabolic signals [7]; consequently to the binding, allosteric changes occur that affect the affinity for partner molecules [8].

HIF-1α and HIF-2α contain an additional N-terminal TAD and an oxygen dependent degradation domain (ODD) that enable HIFs to monitor oxygen concentration. Under normoxia (20% O_2_), two prolines in the ODD are hydroxylated by the HIF prolyl hydroxylases, PHD1–3, and recognized by the von Hippel–Lindau (VHL) tumor suppressor protein, the substrate-binding subunit of the E3 ubiquitin ligase complex. The binding causes polyubiquitination and targets HIF-α to the proteasome [1]. In hypoxic conditions, HIF-α escapes degradation and, after translocation to the nucleus, heterodimerizes with ARNT, binds hypoxia response elements (HREs) in the enhancer regions of target genes, interacts with CBP-p300 complex and initiates transcription [1]. Activated genes are involved in glycolysis, erythropoiesis and angiogenesis; the gene products include erythropoietin, that stimulates the production of red blood cells, and vascular endothelial growth factor (VEGF), a regulator of blood vessel growth [1].

In tumor masses, the abnormal vasculature creates hypoxic regions that activate HIFs to promote angiogenesis and to switch to anaerobic metabolism, sustaining cell viability under hypoxic conditions [9]. HIFs are commonly upregulated in a broad range of cancers [10–12] where they contribute also to resistance to oxidative stress, epithelial–mesenchymal transition (EMT), and tumor invasiveness. HIF-1α and HIF-2α accumulation can also be caused by reduced degradation, as in VHL syndrome, an inherited familial cancer syndrome where mutation of VHL causes its inactivation [13].

Internal hydrophobic cavities are observed in all available structures of bHLH-PAS family within both their PAS-A and PAS-B domains [14]. AhR uses its PAS-B internal cavity for binding a diverse set of small molecules thus activating nuclear translocation, dimerization with ARNT and DNA binding [15,16]. In other PAS domains, ligand binding in the pockets induces long distance conformational changes that affect protein-protein interactions [17], suggesting that PAS cavities can contain allosteric sites [7,18]. In addition to the PAS domain cavities, HIF-1α:ARNT and HIF-2α:ARNT structures show a pocket at HIF-α PAS-B:ARNT PAS-A interface, which has been targeted by acriflavin [19], a mix of trypaflavin and proflavin that acts as a potent inhibitor of both HIF-1α and HIF-2α dimerization with ARNT in cells [20].

As HIF-α:ARNT dimerization is essential to bind DNA and initiate transcription, destabilizing protein–protein interactions in this system represents an optimal therapeutic approach for tumor treatment. However, while direct antagonizing of the interfaces with small molecules is pharmacologically demanding and often unsuccessful due to the troublesome identification of the key residues to target [21], exploiting PAS internal cavities offers potential advantages, especially in terms of selectivity. The need of developing isoform-specific drugs emerges from the observations that HIF-1α and HIF-2α target distinct genes [4], in some cases affecting tumor progression in opposite ways [11]. HIF-2α PAS-B domain contains a relatively large (290 Å^3^) cavity that can be occupied by either water or small molecules with sub-micromolar affinities. These small binders have been shown to impair heterodimerization of isolated PAS-B domains in vitro [22,23]. In the framework of extensive efforts directed to identify inhibitors of HIF-2α:ARNT dimerization [24,25], a molecule has been recently developed, 0X3 (N-(3-chloro-5-fluorophenyl)-4-nitro-2,1,3-benzoxadiazol-5-amine), that is able to disrupt heterodimerization also in living cells [26]. The compound fails to bind to HIF-1α, which has a smaller cavity in PAS-B domain. Albeit the molecular details of 0X3 interaction with HIF-2α PAS-B have been unveiled, how the ligand binding destabilizes the HIF-2α:ARNT complex remains unexplained. The recently determined structures of the entire bHLH-PAS region of the HIF-2α:ARNT dimer in the unbound, DNA-bound, and inhibitor-bound (0X3 and proflavin ligands) forms [19] provide a sound basis for assessing the inhibition mechanism. These dimer structures show that, while the two bHLH domains become linked in a pseudo-symmetric arrangement, the PAS domains interact asymmetrically. Besides the interfaces between corresponding PAS domains, there are also interfaces formed by HIF-2α PAS-B-ARNT PAS-A and HIF-2α intramolecular interactions between PAS-A and PAS-B and between PAS-A and bHLH domains. The lack of physical interaction between ARNT domains facilitates flexibility for arrangements with different partners. Indeed in the NPAS1: and NPAS3:ARNT heterodimers, ARNT PAS-B domain is slightly displaced in comparison with HIF-α:ARNT complex [14]. PAS-B cavity residues facing 0X3 in the HIF-2α:ARNT-0X3 complex are not significantly perturbed, while the PAS domains slightly shifts one respect to the others, with major rearrangements occurring at the interface between the HIF-2α and ARNT PAS-B domains [19].

Here we hypothesized that the ligand-induced local perturbation at the HIF-2α PAS-B domain dynamically propagates through the HIF-2α:ARNT dimerization interfaces by an allosteric inhibition mechanism. To study the functional dynamics of the complex and shed light into the mechanism of regulation of dimer stability, we compared the evolutionary, dynamical and energetic properties of HIF-2α:ARNT dimer structure in its unbound and 0X3-bound form. We identified both the molecular features of the ligand-induced perturbation and the key residues involved in inter-domain communication paths. This novel insight in HIF-2α regulation will guide the development of new specific inhibitors of aberrant HIF-2α activity.

## Methods

### ConSurf analysis

Mapping of residue evolutionary conservation on protein structure can provide reliable prediction of functionally relevant elements and their role in multi domain organisation [27]. Residue conservation on protein surfaces was analysed with ConSurf [28,29]. PAS-domain sequences were detected with a PSI-BLAST [30] search (3 iterations; E-value cutoff 0.0001) of the PDB sequence of HIF-2α:ARNT (PDB ID: 4ZP4) against the UniProt database [31]. Orthologous sequences were manually selected for each protein independently: HIF-1α, HIF-2α and HIF-3α for HIF-2α, and ARNT1 and ARNT2 for ARNT, for a total of 110 and 170 sequences (S1 Table). Input multiple sequence alignments were generated with Muscle [32].

### System preparation and molecular dynamics simulations

Crystal structures for HIF-2α:ARNT dimer in its apo (PDB ID: 4ZP4) and holo (PDB ID: 4ZQD) forms [19] were obtained from the Protein Data Bank (PDB)[33]. The structures have five unresolved segments on each partner: two inter-domain (bHLH/PAS-A and PAS-A/PAS-B) linkers and three intra-domain PAS-A loops. Among these unresolved segments, the GH loop and the PAS-A/PAS-B linker of HIF-2α are available in at least another crystal structure. The other eight unresolved segments in the apo deposition were modelled using the Rosetta all-atom *de novo* loop modelling method with the Next Generation Kinematic closure (NGK) procedure, a variant of the Kinematic Closure (KIC) approach [34–36]. A starting set of 1000 loop models was generated with the parameters proposed in [37], enabling the Taboo sampling feature and using Monte-Carlo simulated annealing for rotamer-based side-chain optimization in a neighborhood of 10 Å around the loop structures. The ensemble of models was then clustered by backbone structural similarity using the Self Organizing Map (SOM) approach previously described in [38–40]. The best conformation for each loop was selected from the most populated cluster by Rosetta energy score. Missing regions in the holo structure were completed by grafting the atomic coordinates of the loops modelled for the apo form and refined using Modeller 9v8 [41]. The completed structures were then pre-processed for simulation with the Schrodinger's Protein Preparation Wizard tool [42]: hydrogen atoms were added, all water molecules were removed, C and N terminal capping were added, disulfide bonds were assigned and residue protonation states were determined by PROPKA [43] at pH = 7.0. Each system was then solvated in an octahedral box with TIP3P water molecules, and neutralized with Na^+^ ions using the GROMACS [44] preparation tools. The minimal distance between the protein and the box boundaries was set to 12 Å. Simulations were run using GROMACS 5.1 [44] with Amber ff99sb*-ILDNP force-field [45]. 0X3 inhibitor in the holo structure was parameterised using GAFF [46]. 0X3 charges were calculated with the restricted electrostatic potential (RESP) method [47] at HF/6-31G* after *ab-initio* optimization of the ligand. A multistage equilibration protocol (modified from [48]) was applied to all simulations to remove unfavourable contacts and provide a reliable starting point for the production runs: the system was first subjected to 1000 step of steepest descent energy minimization, followed by 1000 step of conjugate gradient with positional restraints (2000 kJ mol^-1^ nM^-2^) on all resolved atoms. This minimization process was then repeated with weaker (1000 kJ mol^-1^ nM^-2^) restraints on the backbone of resolved regions. Subsequently a 200 ps NVT MD simulation was used to heat the system from 0 to 100 K with restraints lowered to 400 kJ mol^-1^ nM^-2^ and then the system was heated up to 300 K in 400 ps during a NPT simulation with further lowered restraint (200 kJ mol^-1^ nM^-2^). Finally, the system was equilibrated during a NPT simulation for 1 ns with backbone restraints lowered to 50 kJ mol^-1^ nM^-2^. All the restraints were removed for the production runs at 300 K. A set of three production replicas of 300 ns each were performed for the apo and holo forms. In the NVT simulations temperature was controlled by the Berendsen thermostat [49] with coupling constant of 0.2 ps, while in the NPT simulations the V-rescale thermostat [50] (coupling constant of 0.1 ps) was used and pressure was set to 1 bar with the Parrinello-Rahman barostat [51] (coupling constant of 2 ps). A time step of 2.0 fs was used, together with the LINCS [52] algorithm to constrain all the bonds. The particle mesh Ewald method [53] was used to treat the long-range electrostatic interactions with the cutoff distances set at 12 Å.

### Analysis of geometrical properties

The dynamics of the HIF-2α:ARNT dimer both in the unbound and 0X3-bound forms was investigated to characterise the flexibility of the inter-domain interfaces. Global structural changes during the simulations were monitored by Root Mean Square Deviation (RMSD) from the initial structure. RMSD values were calculated after best fit superposition on the protein Cα atoms. Average per-residue flexibility was measured by RMSF of the atomic positions. RMSD and RMSF values were calculated for the protein Cα atoms using the R [54] Bio3D package [55], both for complete dimer and core domains excluding linkers and loops. All RMSF values were computed on a trajectory obtained concatenating the three replicas, excluding the first 50 ns of each simulation. Secondary structure attribution was done with DSSP [56]. Cluster analysis of the inhibitor geometries in the binding pocket was performed using the GROMOS nearest neighbour algorithm [57] implemented in GROMACS analysis tools, after fitting on the Cα atoms of HIF-2α PAS-B domain. The occurrence during the simulations of water-mediated interactions between HIF-2α residue S304 and 0X3 ligand nitro group was evaluated using GROMACS analysis tools. H-bond detection was done with two sets of thresholds, in agreement with GROMACS (donor-acceptor distance < 3.5 Å and hydrogen-donor-acceptor angle < 30°) and HBplus (donor-acceptor distance < 3.9 Å and hydrogen-donor-acceptor angle < 90°) [58] criteria.

### Analysis of energetic properties

MD simulations of the unbound and 0X3-bound HIF-2α:ARNT dimers were also analysed to identify the role of domains and secondary structures in the dimer stability. The binding free energy of dimer formation was estimated using the Molecular Mechanics Generalized Born Surface Area (MM-GBSA) method [59,60], implemented in the AMBER software package [61,62]. In this method, the ΔG_binding_ is obtained as the sum of energy and configurational entropy contributions associated with complex formation in the gas-phase and the difference in solvation free energies between the complex and the unbound monomers. The configurational entropy of the solute can be estimated using various approximations, but its determination remains a challenging task and usually is neglected [63]. In this work, ΔG_binding_ is determined omitting the entropic term and therefore it is referred to as dimerization energy. The method includes an implicit solvent model. The polar solvation term was approximated with the Generalized Born (GB) model [64] using OBC re-scaling of the effective Born radii [65]. The non-polar solvation term was calculated as the product of the surface tension parameter and the solvent accessible surface area (SA) evaluated using the Linear Combination of Pairwise Overlap (LCPO) algorithm [66]. The single-trajectory approach [63] was used. In this approach both monomer conformations for the calculation were obtained from the dimerized state MD simulation instead of performing distinct simulations of the three different states (monomeric ARNT, monomeric HIF-2α, and bound state). MM-GBSA calculations were performed on a subset of conformations from the equilibrated part of the MD simulations. For this purpose, the domain contributions were calculated at 100ps frequency on the stable interval (100–300 ns) of each replica and each energy component was determined by averaging over the contributions from all the conformers. Single interfaces were analysed using a per-residue energy decomposition. For this purpose, a common ensemble of conformations sampled in all three replicas was identified in the principal component subspace of inter-domain motions, calculated on the subset of Cα at domain interface (S1 Fig). In this analysis, a residue of a domain A was considered within the A-B interface if at least one of its atoms was found within 3.5 Å from an atom of the B domain in at least 10% of the simulation.

### Analysis of correlated motions and long-distance communication

To identify protein segments with correlated atomic motions, a correlation network analysis [67] was performed using Bio3D [55,68]. Pairwise residue cross correlation coefficients were calculated from the displacement of the Cα atom pairs [69]. A weighted graph was generated from the cross correlation matrix, in which each residue represents a node and an edge is drawn when the absolute correlation between two residues is greater than 0.4. Edges with positive weights connect residues with correlated motion, while negative weights describe anti-correlated motions. Shortest and suboptimal path analysis [68], conducted on the 50 shortest detectable paths, was used to highlight differences in interdomain communication in the apo and holo states. The obtained network contains substructures of nodes, called communities, that are more densely interconnected to each other than to other nodes in the network. This community structure was detected using the random walker algorithm [68] on the edges with absolute correlation values greater than 0.5.

### Analysis of database annotated mutations

The role of residues identified as important for dimer stabilisation or inhibition by computational analysis was validated by comparison with point mutations reported in the literature. Two sets of mutations were considered: 1) selected mutations with experimentally verified effects; 2) missense mutations from whole-exome sequencing analysis of different tumor tissues annotated in the COSMIC v83 database [70] for HIF-2α and ARNT. The impact of the mutations in the latter set was predicted using two online webservers: Polyphen2 [71] and SIFT [72]. These webservers estimate the impact of amino acid substitutions based on degree of conservation, physical and evolutionary properties. Mutations were considered ‘benign’ if indicated as such by either one of the two webservers, ‘probably damaging’ if Polyphen2 score was greater than 0.95 and SIFT score was between 0–0.01 with a Median Information Content ≥ 2.6; in all other cases mutations were considered ‘possibly damaging’.

### Data analysis and visualisation

Sequence alignment of ARNT and HIF-2α was obtained by Clustal Omega [73] and visualised using the ESPript server [74]. Protein structure representations were generated with PyMol [75].

## Results

### The domain structure of HIF-2α:ARNT dimer

**Fig 1:**
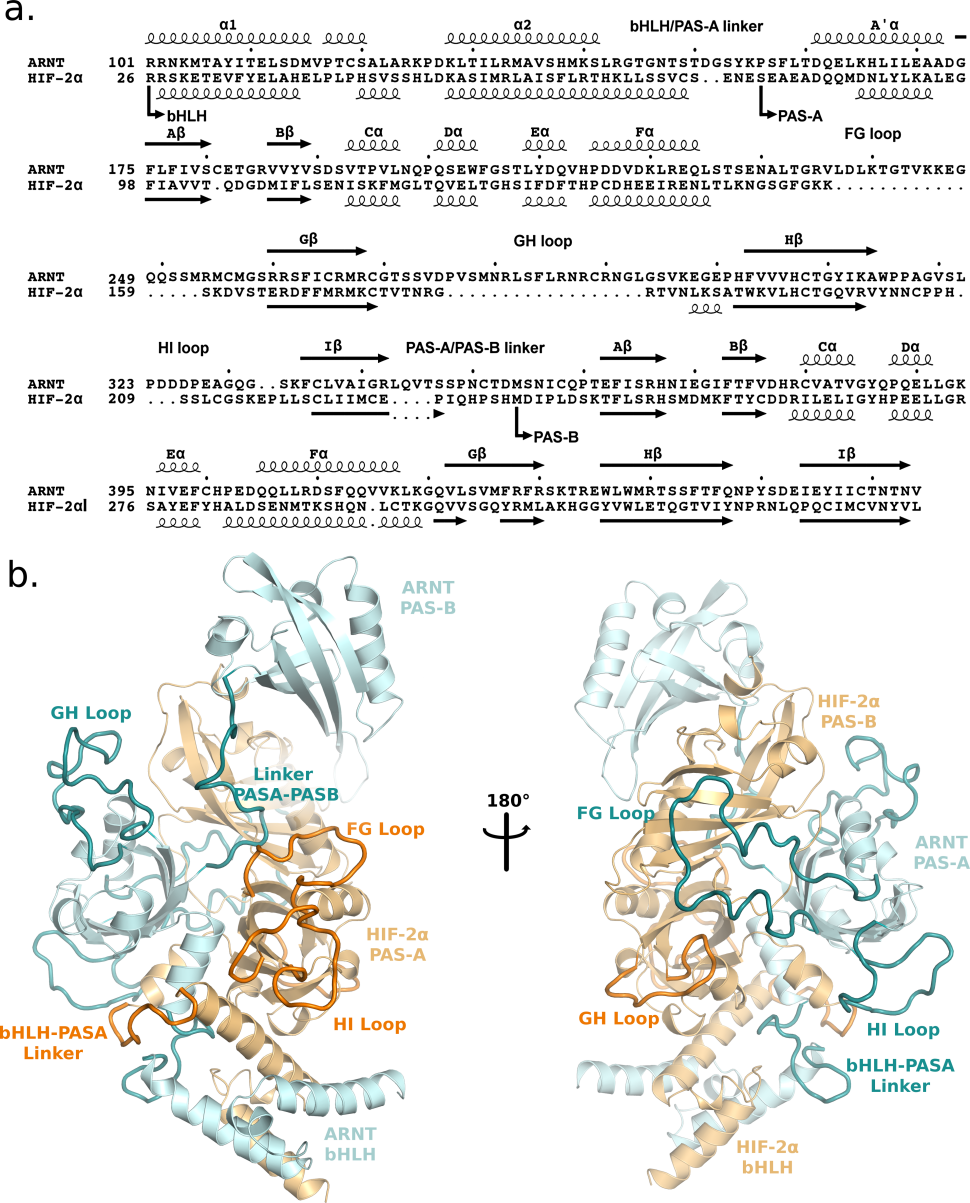
Sequence and structure of the HIF-2α:ARNT dimer. a. Domains and secondary structures of ARNT and HIF-2α in the bHLH-PAS region. Secondary structures were provided by DSSP on the PDB file 4ZP4: helices are displayed as squiggles and β-strands as arrows, and labelled according to the PAS domain nomenclature [18]. b. Cartoon representation of the unbound dimer structure (4ZP4) with the modelled loops and linkers: ARNT in cyan, HIF-2α in orange. Modelled segments are represented in darker colours.

Domains and secondary structures for the two protomers of the HIF-2α:ARNT dimer [19] are presented in Fig 1A. Each protomer includes three domains (bHLH, PAS-A and PAS-B). An overall view of the complex structure, including the modelled loops, is shown in Fig 1B. The dimer has a compact core region formed by ARNT-PAS-A, HIF-2α-PAS-A and HIF-2α-PAS-B domains; this is connected to the bHLH region of both the dimerization partners on one side and the ARNT-PAS-B domain on the opposite side.

**Fig 2:**
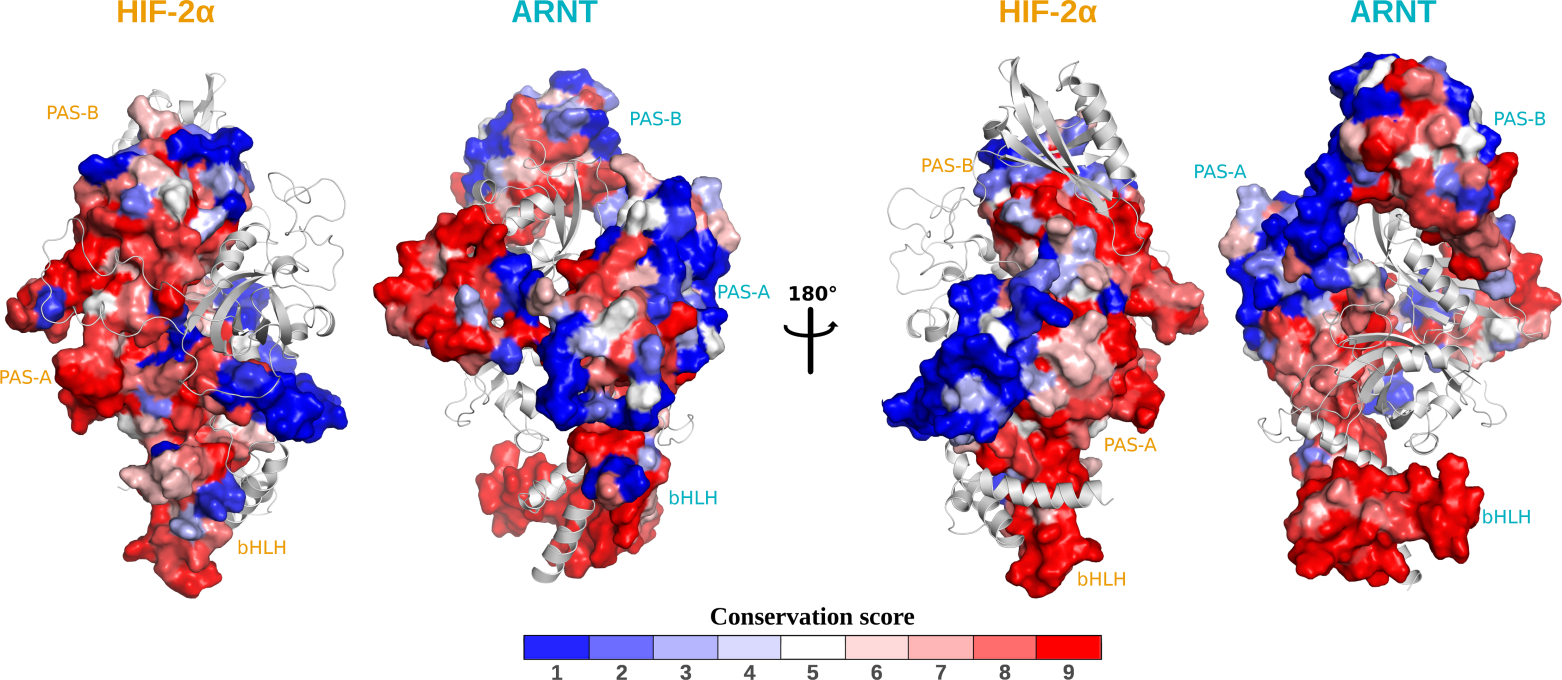
Residue evolutionary conservation mapped on the solvent accessible surfaces. In each representation, the solvent accessible surface is shown for one protein, while the protein partner is depicted in light-grey coloured cartoons. Protein domains are labelled in different colours (ARNT: cyan, HIF-2α: orange). Evolutionary conservation scores (ConSurf ranges: 1 poorly conserved, 9 highly conserved) are reported in a blue-white-red colour scale.

ConSurf conservation profiles highlight highly conserved patches on the bHLH and PAS core domains, especially for the residues lying at the dimerization interfaces (Fig 2 and annotated sequences in S2 Fig).

As expected, the most conserved region is the bHLH domain responsible for DNA binding, while loops are generally poorly conserved. It should be noted that most of the modelled loops belonging to the PAS-A domains resemble structural embellishment with a typical Ω-loop shape and no expected functional role. On the contrary, the medium to high conservation observed for many residues belonging to the ARNT-PAS-A FG loop, the HIF-2α-PAS-A GH loop and the HIF-2α-PAS-A/PAS-B linker (S2 Fig) suggests that these elements may have a functional role. Also the C-terminal linker of the HIF-2α PAS-B, including a loop and a short α-helix and inserted into the PAS-B:PAS-B interface, shows some conserved residues (S2 Fig), suggesting this connecting element may give a contribution to dimerization.

### Analysis of HIF-2α:ARNT dynamics

The RMSD plots of the core domains and complete system (S3 Fig) show well equilibrated trajectories after 50 ns. High flexibility in the PAS-A loops is evident from the root mean square fluctuation (RMSF) plot of the concatenated trajectories for the complete system (S4 Fig), while the bHLH regions show enhanced flexibility due to their terminal position and lack of DNA interactions. As shown in the previous subsection (see **The domain structure of HIF-2α:ARNT dimer**) ARNT PAS-B domain is involved in fewer interactions with the rest of the complex. In all three 300 ns simulation replicas of the unbound dimer we detected a reorientation of ARNT PAS-B and observed that the domain hinge-bending motion around the PAS-A/PAS-B linker spans the same conformational space sampled by different crystallographic structures of ARNT in complex with HIF-2α and other partners (S5 Fig) [14].

**Fig 3:**
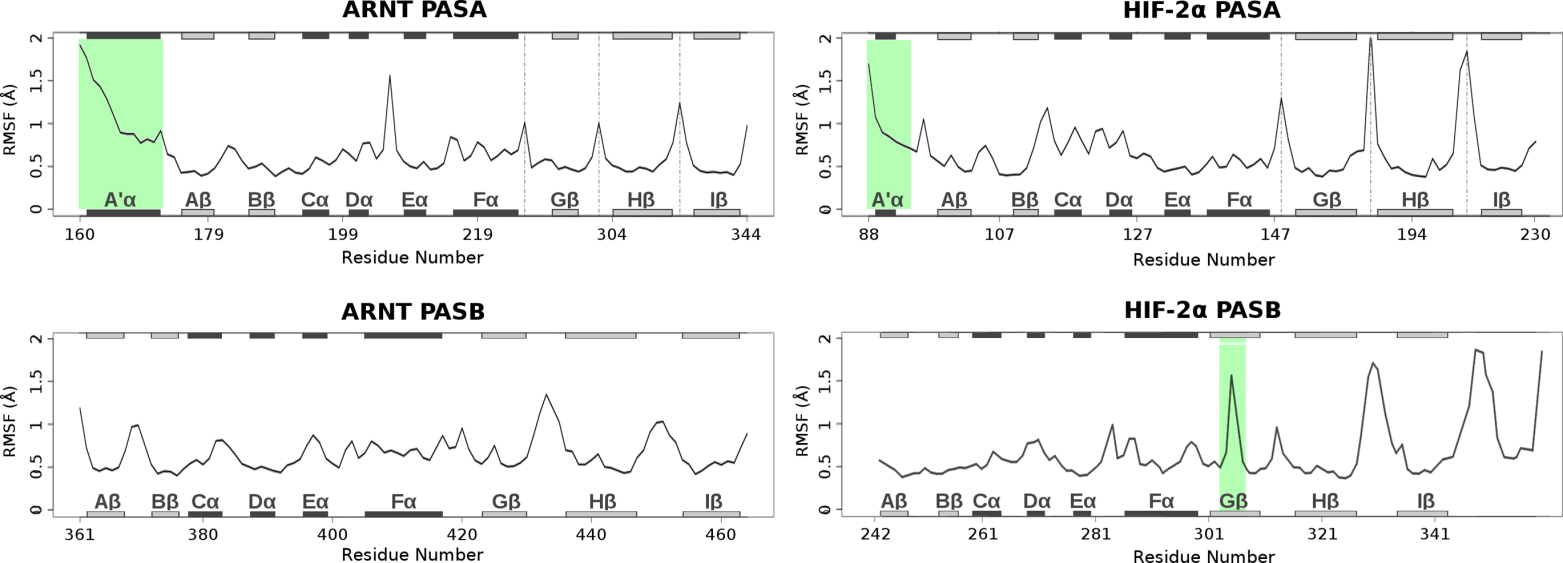
Cα RMSF plots for each PAS domain in the dimer. Structured regions with higher fluctuations are highlighted in green and discussed in text. The long and highly flexible PAS-A loops were excluded from calculation and are indicated by dash lines. Helices and β-strands are represented as black and grey bars, respectively, and labelled according to Fig 1A.

A’ helices of both PAS-A domains show high flexibility (Fig 3), especially in ARNT, while other secondary structure elements are generally rigid, with relatively high RMSF values only in correspondence of connective loops. The only exception is in the HIF-2α PAS-B G-strand, which shows a high degree of flexibility in its central residues. This is specific of the HIF-2α PAS-B domain and is not found in the other PAS domains. Moreover, this β-strand is partially unstable and its central region alternates between unstructured (≈ 70%) and folded (≈ 30%) conformations during the simulations (S6 Fig). This instability of the Gβ strand is consistent with the structures in the NMR ensemble of the isolated HIF-2α PAS-B (PDB ID: 1P97) which contains a completely structured G-strand only in 7 out of 20 states (S7 Fig).

Interface interaction energies were estimated by MM-GBSA. A summarized view of the relative contribution to the dimerization energy provided by each domain is reported in S2 Table. Values were derived as sum of per-residue contributions averaged over the three replicas of 300 ns. The bHLH domains of the two units equally contribute to the stabilization of the dimer. Due to its central position within the quaternary assembly of the dimer (see Fig 1B), the HIF-2α PAS-B domain highly contributes to the dimerization energy, while the ARNT PAS-B domain only interacts with the HIF-2α PAS-B domain, and seems less important in the dimerization. Notably, the HIF-2α C-terminal linker shows a high contribution to the dimerization energy (4.1%) compared to that of the entire HIF-2α PAS-B domain (14.0%). As expected, the dominant role in the dimer association is adopted by the ARNT PAS-A domain (21.0%), that interacts with the bHLH region and with both the HIF-2α PAS domains. A relevant insight arising from the MM-GBSA analysis concerns the importance of ARNT PAS-A FG loop. The 30-residue long loop stands out from the other PAS-A loops for its contribution to dimerization which is at least four times greater than the others (S2 Table). This is due to strong interactions with both the C terminal region of the HIF-2α PAS-A-PAS-B linker and two HIF-2α PAS-B elements, A-strand and C-helix (both highly conserved).

### Analysis of the effect of 0X3 inhibitor on the dynamics of the dimer

Similar to the apo state, the 0X3-bound form has limited flexibility, mostly located in the loop regions. The holo and apo simulations show a quite similar intra-domain RMSF profile for all the domains (S8 Fig). Some differences emerge in the Eα helix of ARNT PAS-B, that is outside the dimerization interfaces, and in the Bβ-Cα region of HIF-2α PAS-A, lying at the PAS-A:PAS-A interface. This latter change in flexibility may indicate a perturbation of protein-protein interactions at this interface. Moreover, significant differences appear on HIF-2α PAS-B domain in the F-helix and G-strand elements, that are in strong contact with the ligand, and thus probably subjected to a local perturbation. Even the HIF-2α PAS-B C-terminal is subjected to rigidification, probably correlated with that of the interacting G-strand.

**Fig 4:**
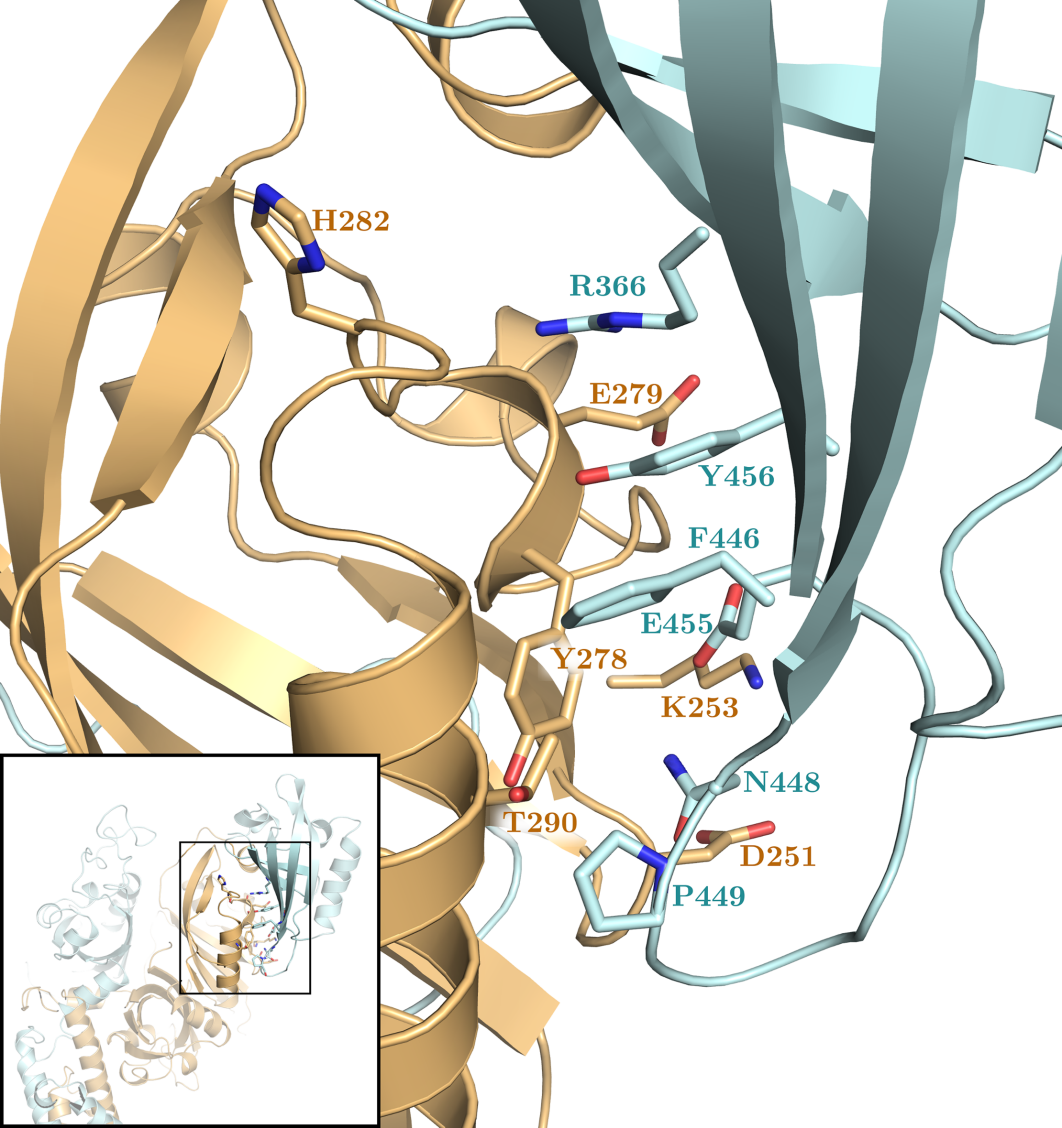
Perturbed dimerization energy at the holo PAS-B:PAS-B interface. 3D representation of the PAS-B:PAS-B interface: ARNT in cyan, HIF-2α in orange. Residues that mostly affect the dimerization energy at this interface in presence of the 0X3 ligand (S3 Table) are shown in sticks.

A comparative analysis of the dimerization energy at the interfaces was done using MM-GBSA. The per-residue decomposition of the dimerization energy highlights a weakening of the interactions at the PAS-B:PAS-B interface in presence of the ligand (S3 Table). The most perturbed region involves key residues for the interface stabilization at both the HIF-2α (from Y278 to T290) and ARNT (from R366 to Y456) side (Fig 4). In details, the perturbation affects the HIF-2α:ARNT electrostatic interactions between E279 and R366, K253 and E455, D251 and N448, as well as the T-stacking between Y278 and F446.

The perturbed region of HIF-2α PAS-B includes residues lying on the E and F helices. As discussed before (S8 Fig), the arrangement of the F-helix appears to be affected by the presence of the ligand, so it is conceivable that this perturbation propagates through the helical bundle, destabilizing the whole PAS-B:PAS-B interface.

**Fig 5:**
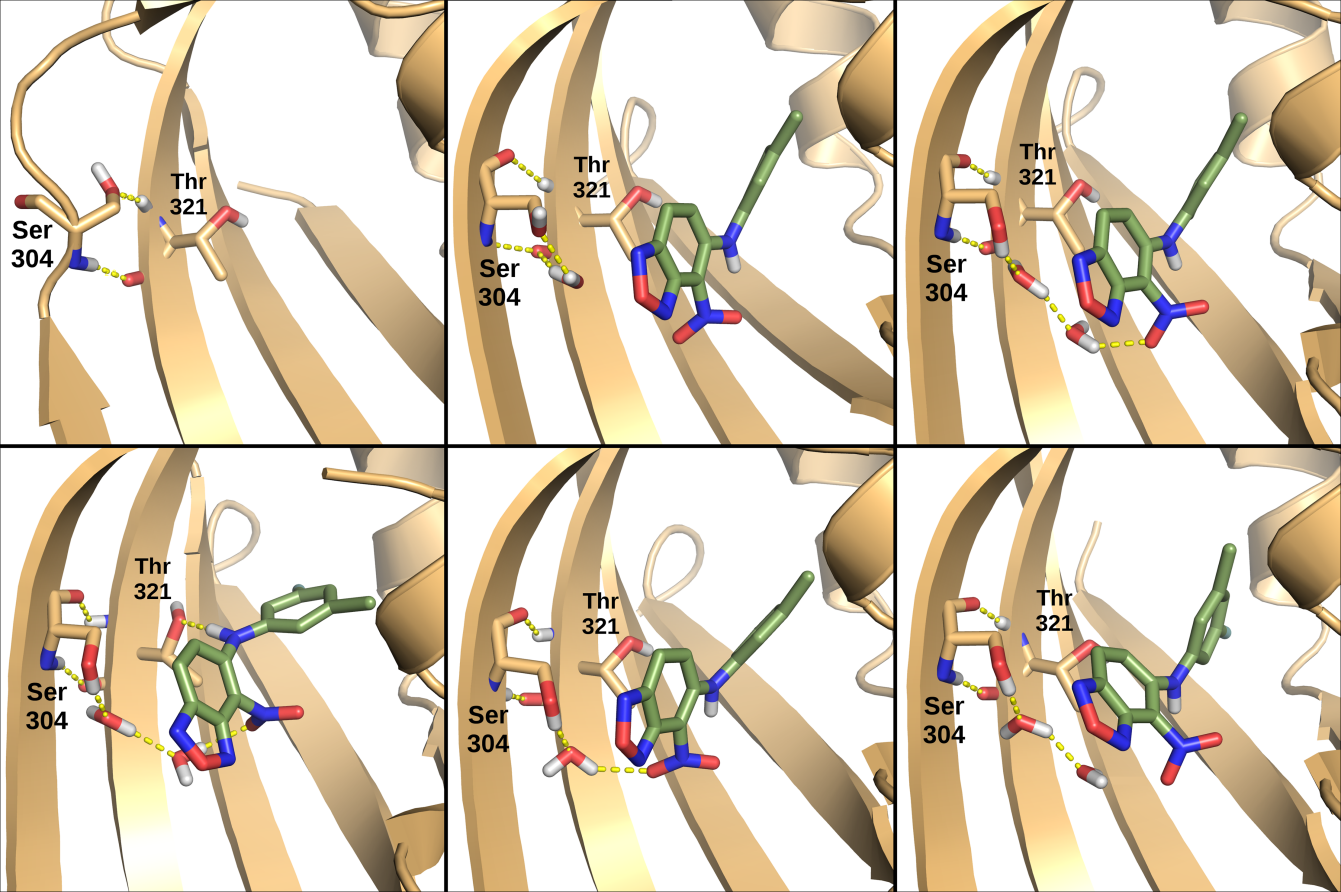
Water mediated hydrogen-bond network between S304 and the 0X3 ligand. In the unbound form (top left panel) the S304 sidechain interacts with the T321 backbone, maintaining this region of the G-strand unstructured. In most of the representative structures of the inhibitor-bound form (remaining panels) the S304 sidechain is involved in a water-mediated hydrogen-bond with the ligand, and the H-bonds between the S304 and T321 backbones facilitate a complete structuring of the strand. H-bond were detected using GROMACS default parameters.

The central part of the HIF-2α PAS-B G-strand is highly flexible and partially unstructured in the apo simulation, while in presence of the 0X3 ligand it is more rigid and fully structured in a β-strand during the entire simulation (S6 Fig). This region of the G-strand includes residues S304, G305 and Q306. S304 is a highly-conserved residue whose side-chain lies within the HIF-2α PAS-B cavity in contact with the ligand. Monitoring of the presence of interactions between S304 and the nitro group of the ligand during the simulations highlighted a stable H-bond network mediated by one or two water molecules (S9 Fig). Overall, interactions occur for about 36.4% of simulation time (21.0% with one water molecule and 16.4% with two water molecules) according to the GROMACS H-bond definition, and 60% of simulation time (28.4% with one water molecule and 31.6% with two water molecules) according to the HBplus H-bond definition. A set of representative ligand conformations (Fig 5) was extracted by cluster analysis and shows different arrangements of these water bridged interactions. The presence of these interactions looks essential for the complete folding of the G-strand.

**Fig 6:**
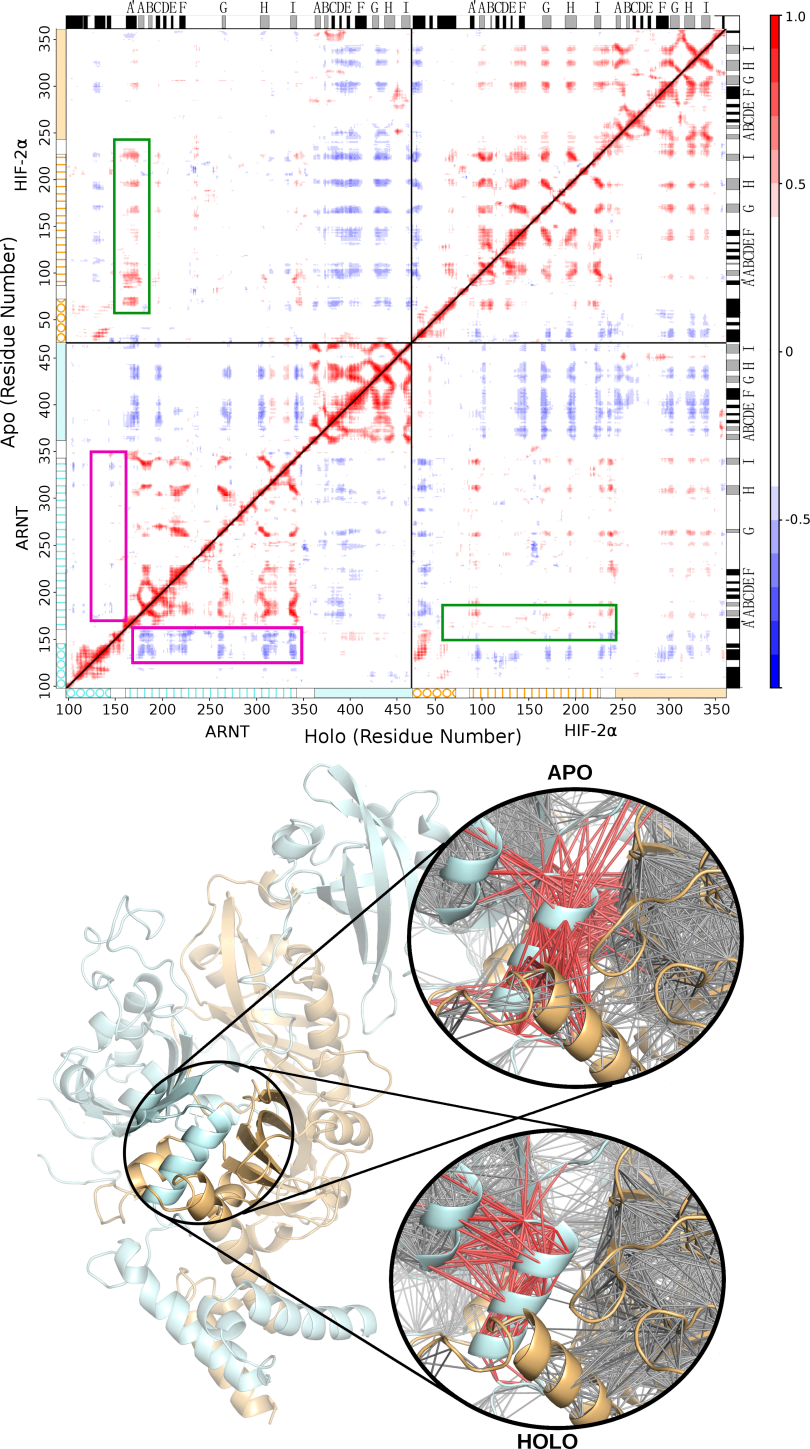
Distance cross correlation matrices for apo and holo simulations. The correlation matrices are shown on the top (upper triangular for apo–lower for holo) with domains (circle = bHLH, vertical lines = PAS-A, light filled = PAS-B) and secondary structure profiles (black = helix, light-grey = sheet) on the sides. A 3D representation of the network derived from the DCCM is represented at the bottom with a close-up of the ARNT PAS-A A’ helix region.

Analysis of residue correlated motions by means of the distance cross correlation matrix [67,69] (DCCM) show long-distance effects of this local perturbation (Fig 6) consistent with a lower stability of the dimer in presence of 0X3.

**Fig 7:**
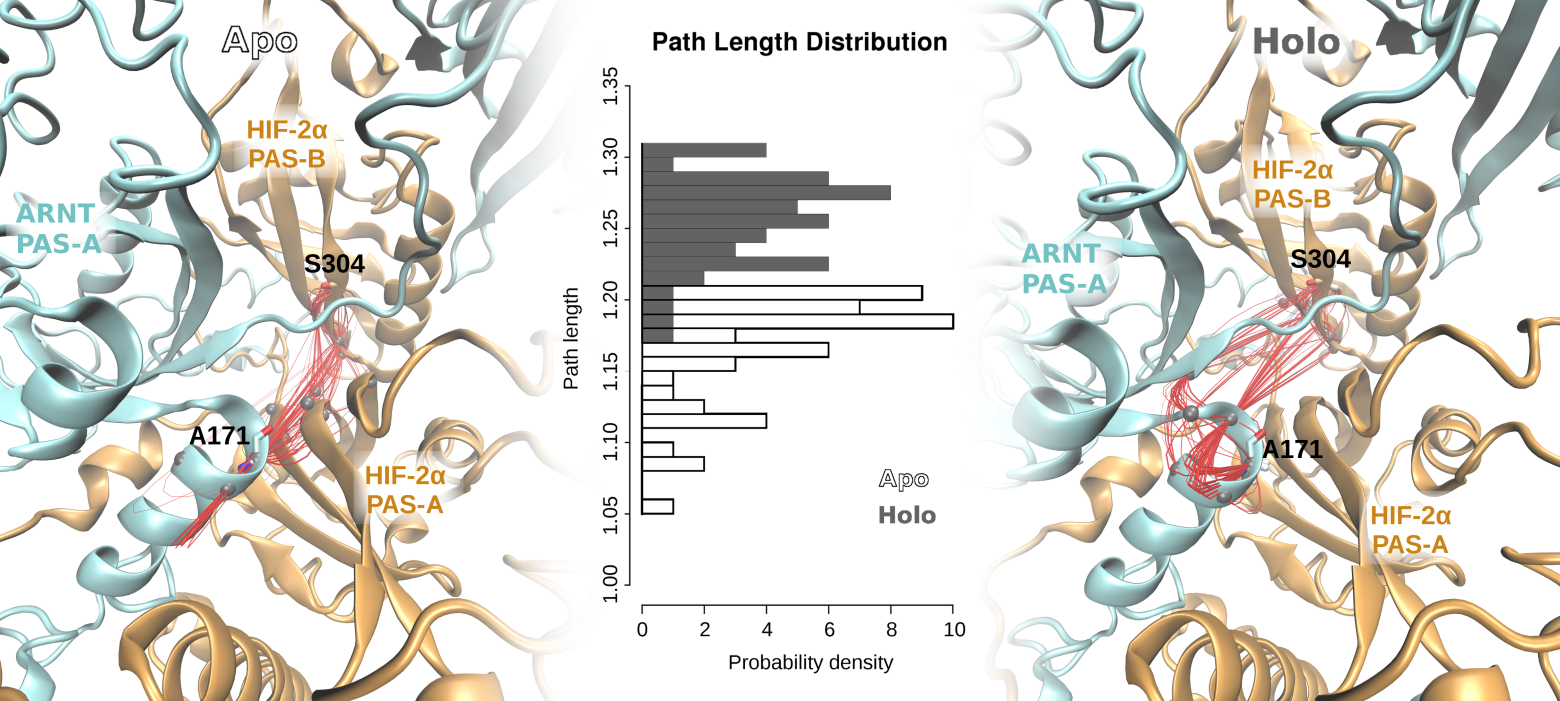
Optimal and suboptimal path analysis for the unbound (apo) and 0X3-bound (holo) HIF-2α:ARNT dimer structures. Left and right panels: paths are shown as red lines connecting residues in the three interface domains (HIF-2α PAS-B and PAS-A in orange cartoon, ARNT PAS-A in cyan cartoon). Central panel: path length distributions between the HIF-2α S304 (in PAS-B) and ARNT A171 (in the A’ helix of PAS-A) residues.

Each domain of the system holds strong internal positive correlation (except for the long PAS-A loops) in both apo and holo simulations, confirming the rigidity of all the PAS domains during the simulations. Major differences are evident in the region of ARNT residues 130–180. In particular, ARNT bHLH-PAS-A linker (magenta squares in Fig 6) has anti-correlated motions towards the ARNT PAS-A domain only in the holo simulation, while in the apo simulation the ARNT PAS-A A’ helix (green squares in Fig 6) is correlated with the HIF-2α PAS-A domain. This suggests that the motion of ARNT PAS-A A’ helix, lying at the PAS-A:PAS-A interface, becomes decoupled from the PAS-A domain after inhibitor binding, probably indicating lower inter-domain interaction. To correlate the altered flexibility of HIF-2α PAS-B G-strand with the perturbed dynamics and correlated motions of ARNT PAS-A A’ helix, we calculated the optimal and suboptimal paths [76] between these two regions from the DCCM networks of the apo and holo simulations (Fig 7).

In the case of the apo network, the shortest paths connect the HIF-2α PAS-B G-strand with the HIF-2α PAS-A strands and then with the ARNT PAS-A A’ helix (Fig 7). In the case of the holo network, the shortest path is altered. For this system, it links the ligand-perturbed HIF-2α PAS-B G-strand to the ARNT PAS-A, implying a longer connection to the A’ helix (Fig 7). The change observed in the communication paths of the apo and holo dimers can also be appreciated by comparing the frequency of each residue occurrence in the best fifty suboptimal paths of the two systems (S10 Fig). Several residues in the HIF-2α PAS-A that participate to these paths with high frequency in the apo system simulation (F168, F169, L193-T196, M225-E227) no longer occur in the holo paths.

A modification in the relative motion of PAS domains after inhibitor binding is also highlighted by a different community structure in the residue correlation network analysis (S11 Fig). Holo simulations are characterised by the independent motion of each single PAS domain. First, a notable change is visible at the PAS-B:PAS-B interface, where ARNT and HIF-2α residues belong to the same community in the apo simulations, while they are separated in domain-specific communities in the holo simulations. Second, residues of HIF-2α-PAS-A:ARNT-PAS-A interface and the HIF-2α PAS-B G-strand are in the same community in the apo simulations, while there is a clear separation by domain in the holo simulations.

## Discussion

Since the discovery of a large cavity within the PAS-B domain of HIF-2α and the identification of compounds that bind this cavity and dissociate HIF-2α from ARNT [22,23,26], several structure-based research programs have been started to find selective and potent antagonists of the HIF-2α transcriptional activity [24,25,77,78]. However, mechanistic understanding of ligand effects on the dimer association had remained elusive until recently, because the available X-ray structures of HIF-2α in complex with artificial ligands encompassed only the isolated HIF-2α and ARNT PAS-B domains. It was first suggested that ligands can induce conformational changes at the PAS-B β-sheet of HIF-2α that weaken the interactions with the ARNT PAS-B β-sheet [24,26], but no evidence in the context of the full dimer was available. Only recently the determination of the crystallographic structures of the entire bHLH-PAS region of the dimer in the apo and 0X3-bound forms [19] has opened the way to a better understanding of the inhibition mechanism. The proximity of 0X3 to the α-helices region of the HIF-2α PAS-B domain, as well as the small perturbation observed in the X-ray structure of the 0X3-bound dimer at this interface, supported a model in which ligand binding could influence the heterodimer stability through a perturbation of its PAS-B:PAS-B interface [19]. However, deeper insight in the atomistic details of this perturbation is limited by the static view provided by the crystallographic structure. Indeed, it is conceivable that 0X3 perturbation could propagate through the structure and affect other interfaces thanks to the dimer intrinsic dynamics and in agreement with a previously suggested allosteric inhibition mechanism [19,26]. To investigate this hypothesis, we characterised the evolutionary, dynamical and energetic properties of the dimerization interfaces in the apo and 0X3-bound form. The results shed light on the atomistic details of 0X3 inhibition mechanism, on the residues involved in dimer stabilisation and on pharmacophoric features required for future development of analogues of 0X3.

**Fig 8:**
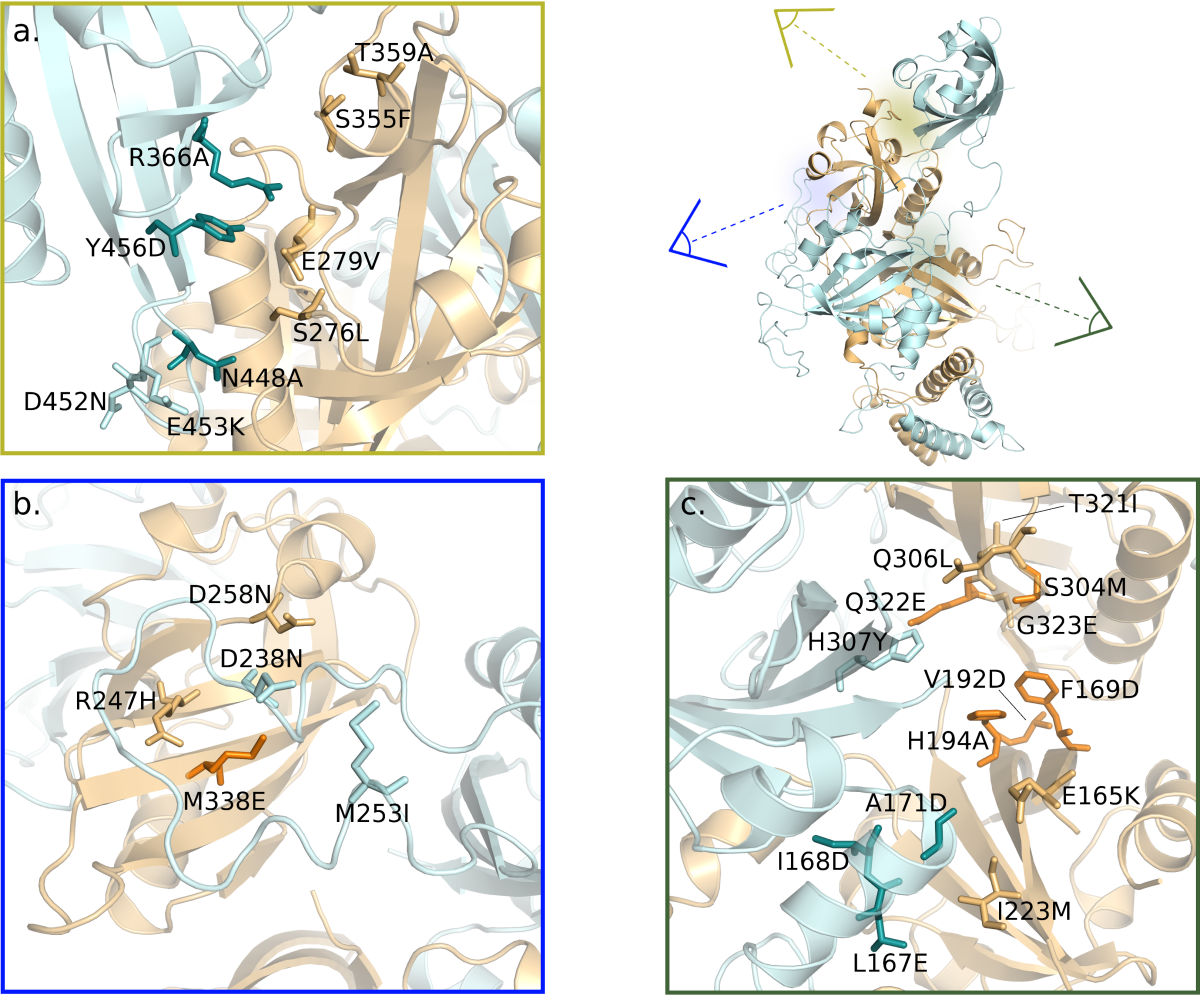
Mutations in the predicted key regions for HIF-2α:ARNT dimerization and inhibition. In the 3D representations of the dimer (ARNT in cyan, HIF-2α in orange), point mutations are labelled and shown in sticks (darker colours for experimental mutations and lighter colours for cancer-related mutations). Upper-right panel: overview of the dimer structure with the location of the three regions here discussed. Close-up panels: a. ligand-perturbed PAS-B:PAS-B interface; b. ARNT PAS-A FG-loop region involved in dimer stabilization; c. ligand-perturbed HIF-2α PAS-B G-strand and inter-domain region (HIF-2α-PAS-B—HIF-2α-PAS-A—ARNT-PAS-A).

Our modelling predictions were tested against a wide set of relevant mutagenesis data (reported in Fig 8 and S4 Table). Several experimental mutations that were proved to destabilize the HIF-2α:ARNT dimer and homologous systems were reported in the recent literature [19][14][79][80][81]. Furthermore, it is known that HIFs function can be affected by mutations that have been observed in different carcinomas, brain gliomas, and skin melanomas [82]; in-depth analysis within the COSMIC database, performed in this work and by other Authors [19] indicated that many cancer-related missense mutations are located in the PAS-A-PAS-B regions at the HIF-2α:ARNT interface, thus offering a valid reference point to validate our hypotheses.

Our residue conservation analysis (Fig 2) detected high scoring patches on all inter-domain interfaces confirming homomeric and heteromeric interactions in agreement with the crystallographic structure [19]. In addition, our results highlighted strong conservation in some connecting elements, thus suggesting their functional role: the HIF-2α-PAS-A GH loop, which involvement in DNA binding was previously demonstrated [19]; the HIF-2α-PAS-A/PAS-B linker, which participation to the ARNT-PAS-A:HIF-2α-PAS-B interface indicates its role in the dimer stabilization; and the ARNT-PAS-A FG loop that we suggest may be involved in the ARNT flexible arrangement around HIF-2α and different partners. Past mutagenesis and co-immunoprecipitation (co-IP) studies indicated that the bHLH:bHLH, PAS-A:PAS-A and HIF-2α PAS-B:ARNT PAS-A interfaces are critical for dimer stability [19]. We assessed this by calculation of the contributions provided by each domain and secondary structure element to the dimerization energy. We confirmed the importance of the bHLH and PAS-A domains of both partners and of the HIF-2α PAS-B domain in the dimer stabilization (S2 Table) and we showed that the ARNT-PAS-A domain gives the major contribution to binding. In addition, we highlighted that the dynamic behaviour of the ARNT-PAS-A FG loop enhances the domain intermolecular interactions by wrapping around the HIF-2α PAS-B domain and that the C-terminus of the HIF-2α PAS-B contributes to the PAS-B:PAS-B interface stabilization. Evidences of the above predictions were found in cancer-related mutations located in the ARNT PAS-A FG loop (in particular, D238N) and in the HIF-2α faced strands (R247H, D258N, Fig 8B and S4 Table), as well as in the short α-helical region of the HIF-2α C-terminal linker (S355F, T359A, Fig 8A and S4 Table).

From MD simulations we detected a general internal rigidity in the PAS domains of both partners, except for the PAS-A loops. This suggests that the dynamics of the system mainly involves quaternary structure oscillations. These motions are particularly evident for the ARNT PAS-B domain, that gives few interactions with the rest of the dimer, and shows characteristic hinge-bending motions around the flexible PAS-A/PAS-B linker. The dynamics of this domain, along with its arrangement (S5 Fig) in the crystallographic structures of ARNT in complex with different bHLH-PAS class-I partners (HIF-1α, HIF-2α, NPAS1 and NPAS3) [2,3,14,19], supports a general model for ARNT dimerization in different heterodimers: strong interactions at the dimerization interfaces in the bHLH/PAS-A region stabilize the dimerization, while the domain bending motion of ARNT PAS-B provides adaptation to different partners through different dimerization geometries in the PAS-B:PAS-B region.

We compared the dimer dynamics in the apo and 0X3-bound forms and identified the dimerization interfaces that are mainly affected by ligand binding as well as the ligand-induced perturbations on intra-domain correlated motions and on inter-domain communication paths. The holo dimer has reduced flexibility in the Eα-Fα region of the HIF-2α PAS-B domain and weakened residue interactions in the PAS-B:PAS-B interface (Fig 4 and S3 Table). This behaviour is in agreement with the hypothesis of Wu and co-workers about the involvement of this interface in dimer inhibition, supported by mutagenesis and co-IP experiments on the HIF-2α:ARNT dimer (ARNT R366A, N448A, Y456D, Fig 8A and S4 Table) [19] as well as on ARNT dimers with different bHLH-PAS class I partners [19][14]. We found additional confirmation by several cancer-related mutations that lie at both sides of the PAS-B:PAS-B interface (in particular the HIF-2α mutations S276L and E279V, Fig 8A and S4 Table). However, in the holo simulations we also detected a previously undescribed perturbation on the opposite side of the HIF-2α PAS-B domain: the G-strand, which is flexible and partially unstructured in the apo simulation, becomes more rigid and fully structured in a β-strand in the presence of the ligand. This regularisation of the strand is triggered by water-bridged interactions of the 0X3 nitro group with S304 sidechain (Fig 5). Previous studies on the isolated HIF-2α PAS-B domain have demonstrated that, among a number of artificial ligands, the ones with a nitrobenzoxadiazole group connected to aromatic/heterocyclic rings by a amine linker, like 0X3, show the highest binding affinities and inhibition potency [24]. While the heterocycle and the nitro group in this molecular moiety were suggested to contribute to high affinity through favourable electrostatic interactions with a few side-chains in the PAS-B binding cavity [24], no clear explanation was provided for their role in the inhibition mechanism. On the other hand, a critical role of water molecules in the stabilisation of the apo cavity was previously reported [83], but our insight on the dynamics of the bound form explains for the first time the atomistic details of 0X3 perturbation mediated by water. Here we propose that the local effect of the inhibitor propagates through HIF-2α-PAS-B interfaces with other domains toward the core dimerization region. This is evident in the perturbed flexibility of the Bβ-Cα region of HIF-2α PAS-A (S8 Fig) and in the change of correlated atomic motions between HIF-2α and ARNT domains (Fig 6). The DCCM network analysis showed that a communication path connecting HIF-2α-PAS-B—HIF-2α-PAS-A—ARNT-PAS-A is present in the apo but lost in the holo simulations (Fig 7). The motion decoupling induced by the loss of this communication is consistent with a weakening of the HIF-2α-PAS-A:ARNT-PAS-A interaction in the A’ helix key region. This is also confirmed by the change in the community structure of the residue correlation networks (S11 Fig) from the apo to holo form, where in the ligand-bound state the domains segregate in different communities. Our prediction of the inhibition mechanism can be validated on the basis of several experimental evidences (Fig 8C and S4 Table). The ligand-induced local perturbation of the HIF-2α G-strand directly affects S304, whose importance was attested by previous mutagenesis experiments reporting that the S304M mutant is unable to bind 0X3 and other similar ligands [24]. Moreover, another residue in the G-strand, Q306, is known to interact with the proflavin inhibitor that, in a reported crystal structure (PDBID: 4ZPH) [19], is shown to bind outside the PAS-B ligand binding cavity known for the bHLH-PAS proteins [18]. The Q306L tumor-associated mutation, along with others in the adjacent HIF-2α H-strand (*i*.*e*. T321I and G323E, Fig 8C) and in the faced ARNT PAS-A elements, confirm this region as a key point of ligand perturbation. Noticeably, our MD analysis showed that the H-bonds between S304 and T321 in the holo dimer facilitate the structuring of the G-strand (Fig 5). Moreover, in our DCCM network analysis a set of residues in the region 168–227 of the HIF-2α-PAS-A domain (S10 Fig) were only present in the shortest path of the apo simulations and are expected to be critical to sustain the dimer interaction. Indeed three of them have been already shown to be essential by previous mutagenesis and co-IP studies (F169D, V192D, H194A, Fig 8C and S4 Table) [19]. Finally, our prediction of the allosteric destabilization of the PAS-A:PAS-A interaction may be supported both by HIF-2α missense mutations at the PAS-A:PAS-A interface (in particular, I223M) and by three point mutations on the ARNT A’ helix that were demonstrated to strongly affect the stability of ARNT dimers not only with HIF-2α (L167E, I168D, A171D, Fig 8C and S4 Table) but also with HIF-1α, NPAS1 and NPAS3 partners [19][14].

In conclusion, the results here presented support a model of inhibition by 0X3 where both HIF-2α-PAS-B interfaces are destabilised: the helical bundle side interacting with the ARNT-PAS-B β-sheet, and the β-sheet side interacting with both the PAS-A domains. This latter perturbation allosterically propagates to the PAS-A:PAS-A interaction interface thus destabilizing one of the most important region for dimer association. A critical role in the initial induction of these effects is played by water-bridged ligand-protein interactions. This suggests that, in addition to previously identified features of successful inhibition of 0X3 [24,26], future drug design may be targeted to insert functional groups to stabilise water-bridged interactions with the key residues in the HIF-2α-PAS-B G-strand.

## Supporting information

S1 Fig Probability density maps of conformations from the combined MD trajectories. Bins are calculated on the subspace of the first two principal component of motions for HIF-2α PAS-B and ARNT PAS-B. Apo (left panel) and holo (right panel) simulations. The white box contains the most populated bins, that include about 15% of the whole trajectory.
(TIF)

S2 Fig Conservation score profile (blue: low conserved, red: highly conserved) obtained by ConSurf. Secondary structure elements according to DSSP for the 4ZP4 PDB structure are reported above each sequence and labelled according to the PAS domain nomenclature.
(TIF)

S3 Fig RMSD plots for the simulations of the HIF-2α:ARNT dimer in the apo form (PDB ID: 4ZP4). RMSD values are calculated on all Cα atoms (upper panel) or on the bHLH-PAS domains excluding loops and linkers (lower panel). In each panel, the RMSD for the three replicas (R1, R2, and R3) are shown.
(TIF)

S4 Fig RMSF plot for the simulations of the HIF-2α:ARNT dimer in the apo form (PDB ID: 4ZP4). RMSF values are calculated on the Cα atoms. Domains are indicated on the top (ARNT: cyan; HIF-2α: orange; circle: bHLH; vertical lines: PAS-A; light filled: PAS-B) and the protein secondary structure elements according to DSSP are reported at the bottom of the graph (black: α-helix, light grey: β-strand).
(TIF)

S5 Fig Close-up of the ARNT PAS-B structures. NPAS1:ARNT X-ray deposition (PDB 5SY5) is shown in grey; HIF-2α:ARNT X-ray deposition (PDB 4ZP4), in cyan; and three representative states extracted from MD simulations, in transparent cyan. The two complete X-ray structures are shown on the right.
(TIF)

S6 Fig Time evolution of the HIF-2α PAS-B secondary structures assignment in the MD simulations of the unbound (top) and bound (bottom) HIF-2α:ARNT structure. Secondary structure elements were assigned using the DSSP algorithm. The location of the Gβ element is indicated on the right-hand side.
(TIF)

S7 Fig HIF-2α PAS-B secondary structures according to DSSP for the 20 conformers in the 1P97 NMR deposition. The secondary structure elements are labelled on the right according to the PAS domain nomenclature.
(TIF)

S8 Fig Comparison of the RMSF plots for each PAS domain of the HIF-2α:ARNT system in the apo (dashed line) and holo (solid line) simulations. Ligand-perturbed regions discussed in the text are highlighted in light green. The long and highly flexible PAS-A loops are excluded from the calculation, and the corresponding gaps are indicated in the figure by vertical dashed lines. Secondary structure elements according to DSSP are reported on the top and bottom of the graphs (black: α-helix, light grey: β-strand).
(TIF)

S9 Fig Water-mediated interactions between HIF-2α S304 and 0X3 nitro group. Interactions are present for the 36.4% of total simulation time. Red lines indicate an interaction mediated by one water molecule and blue lines indicate an interaction mediated by two water molecules. Geometric parameters for H-bond definition were chosen according to the GROMACS definition.
(TIF)

S10 Fig Comparison of residues in suboptimal communication paths in the apo and holo simulations. The frequency of each residue occurrence in the best 50 suboptimal paths is shown.
(TIF)

S11 Fig Community structure derived from the DCCM network in the apo (left) and holo (right) form. Residue positions are coloured according to the community membership.
(TIF)

S1 Table UniRef codes of the amino acid sequences for ConSurf analysis. (DOCX)

S2 Table Domain decomposition of the MM-GBSA ΔG_binding_ for the apo HIF-2α:ARNT dimer. (DOCX)

S3 Table Comparison of the per-residue decomposition of the MM-GBSA ΔG_binding_ between the apo and holo HIF-2α:ARNT dimers at the PAS-B:PAS-B interface. Contributions that differ by more than 0.5 kcal mol^-1^ are highlighted.
(DOCX)

S4 Table Mutations in the key regions for HIF-2α:ARNT dimerization and inhibition. (DOCX)
