# Supplementary figures and images for: Ligand-induced perturbation of the HIF-2α:ARNT dimer dynamics
Source: PLoS Comput Biol. 2018 Feb 28;14(2):e1006021. doi: 10.1371/journal.pcbi.1006021 (PMC5847239; doi:10.1371/journal.pcbi.1006021)

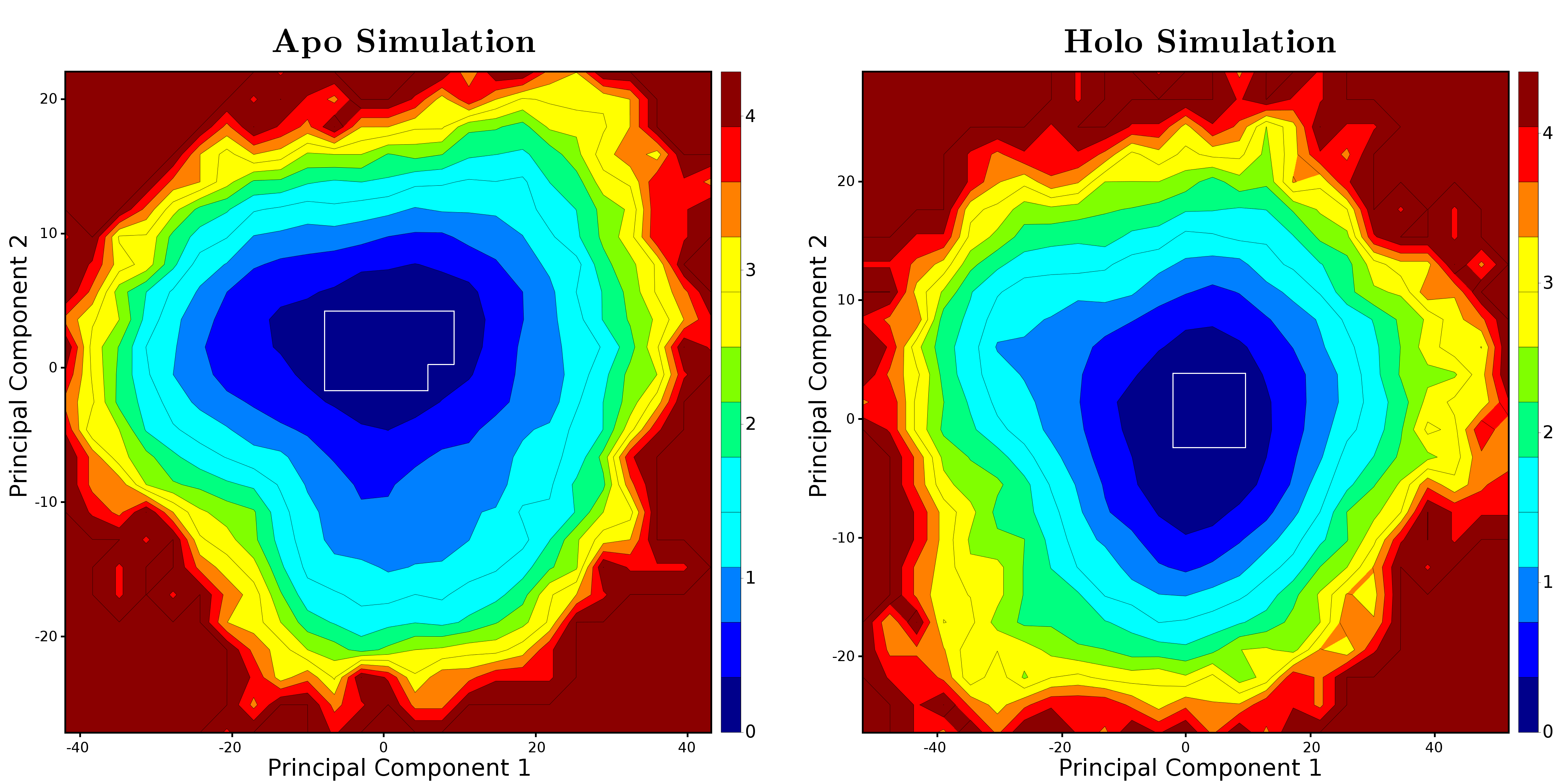

Supplement: S1 Fig — Bins are calculated on the subspace of the first two principal component of motions for HIF-2α PAS-B and ARNT PAS-B. Apo (left panel) and holo (right panel) simulations. The white box contains the most populated bins, that include about 15% of the whole trajectory. (TIF) [file pcbi.1006021.s001.tif]

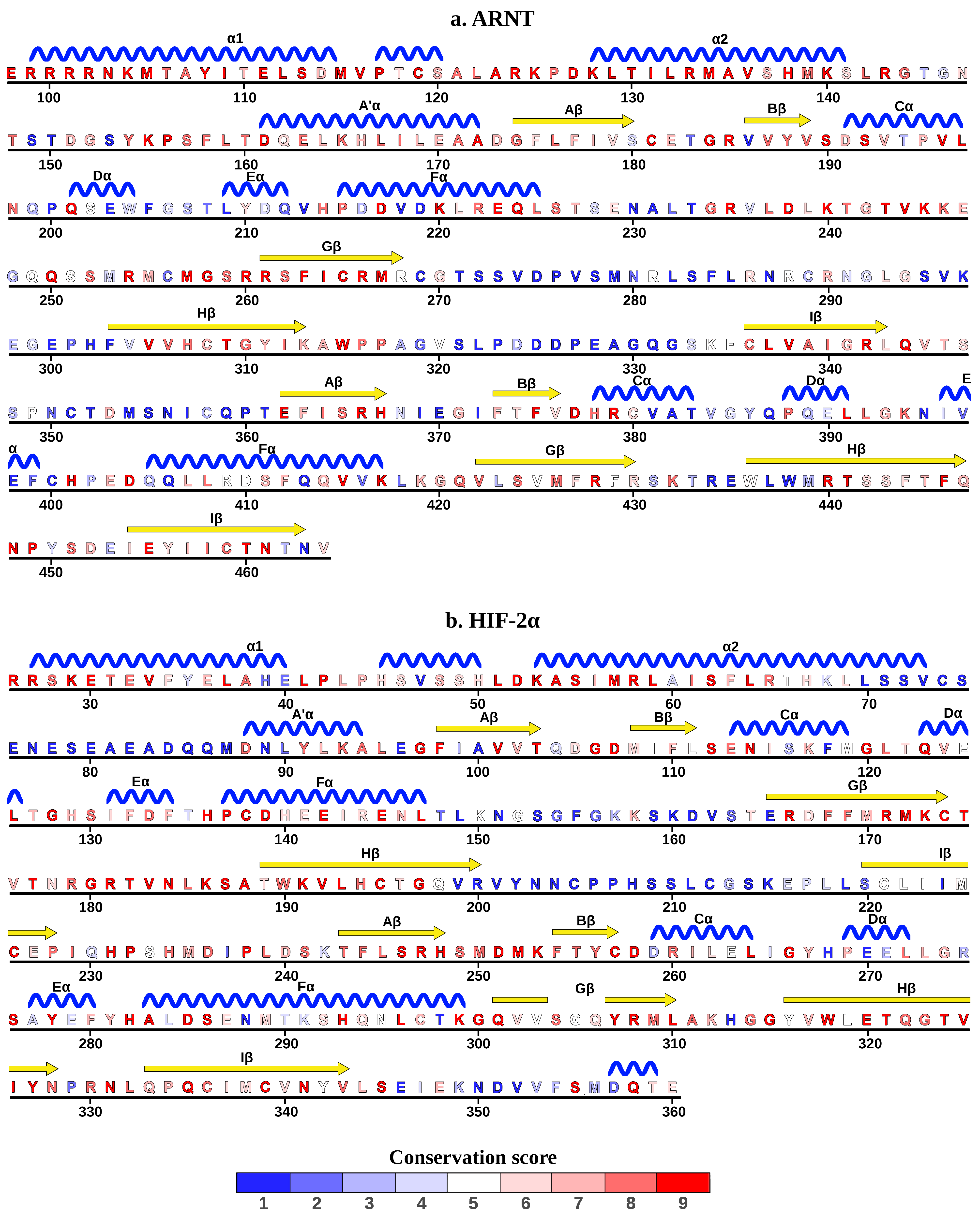

Supplement: S2 Fig — Secondary structure elements according to DSSP for the 4ZP4 PDB structure are reported above each sequence and labelled according to the PAS domain nomenclature. (TIF) [file pcbi.1006021.s002.tif]

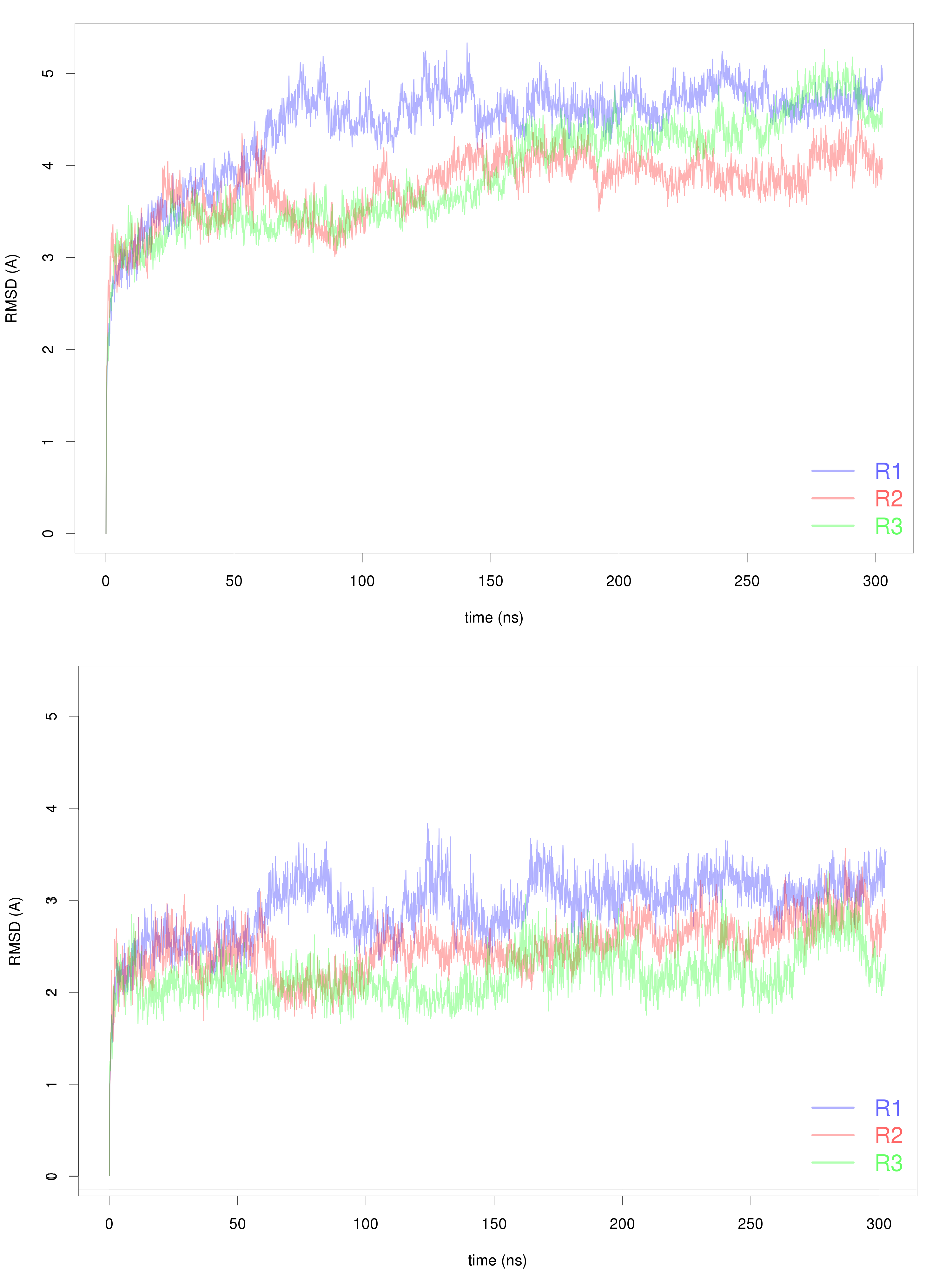

Supplement: S3 Fig — RMSD values are calculated on all Cα atoms (upper panel) or on the bHLH-PAS domains excluding loops and linkers (lower panel). In each panel, the RMSD for the three replicas (R1, R2, and R3) are shown. (TIF) [file pcbi.1006021.s003.tif]

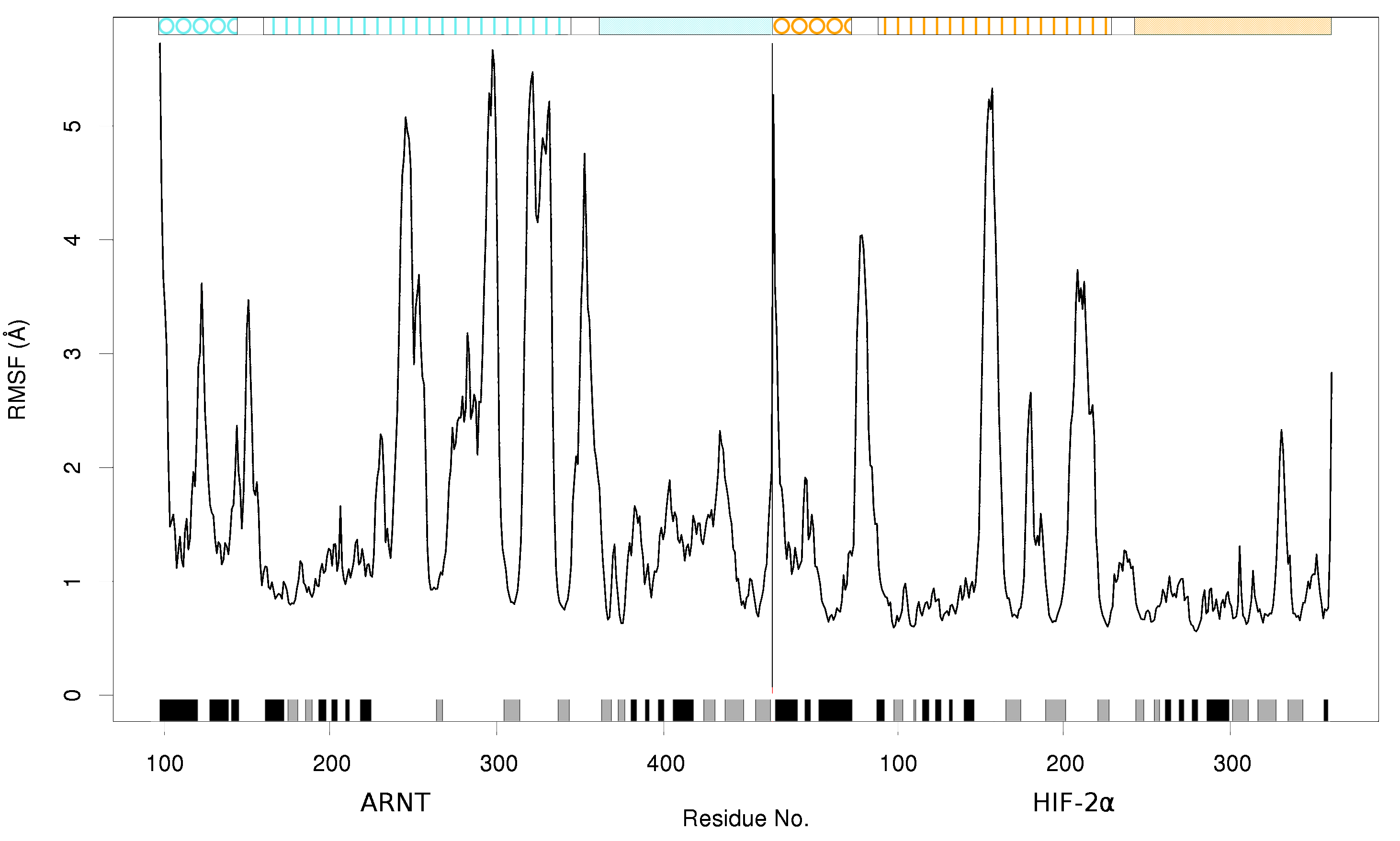

Supplement: S4 Fig — RMSF values are calculated on the Cα atoms. Domains are indicated on the top (ARNT: cyan; HIF-2α: orange; circle: bHLH; vertical lines: PAS-A; light filled: PAS-B) and the protein secondary structure elements according to DSSP are reported at the bottom of the graph (black: α-helix, light grey: β-strand). (TIF) [file pcbi.1006021.s004.tif]

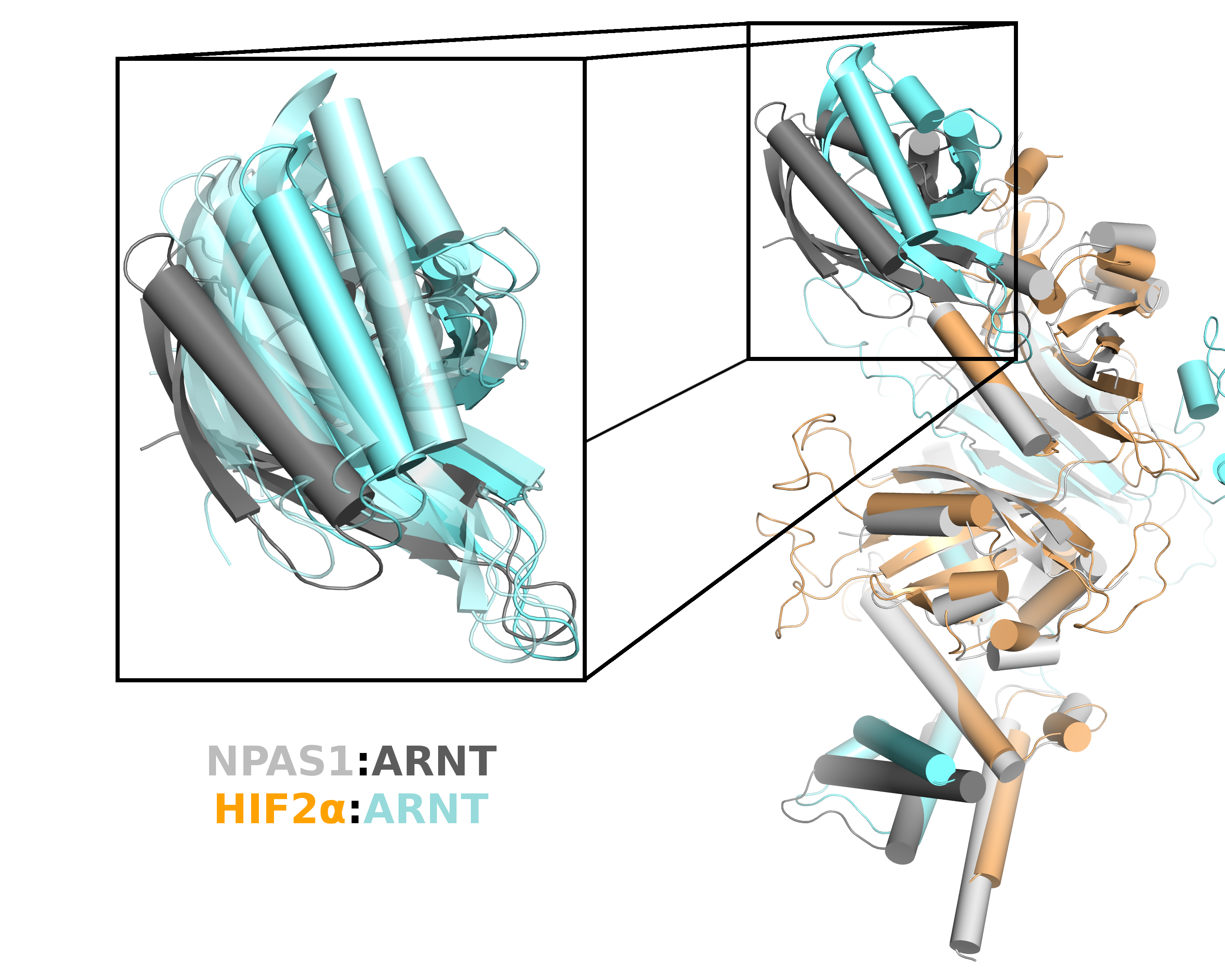

Supplement: S5 Fig — NPAS1:ARNT X-ray deposition (PDB 5SY5) is shown in grey; HIF-2α:ARNT X-ray deposition (PDB 4ZP4), in cyan; and three representative states extracted from MD simulations, in transparent cyan. The two complete X-ray structures are shown on the right. (TIF) [file pcbi.1006021.s005.tif]

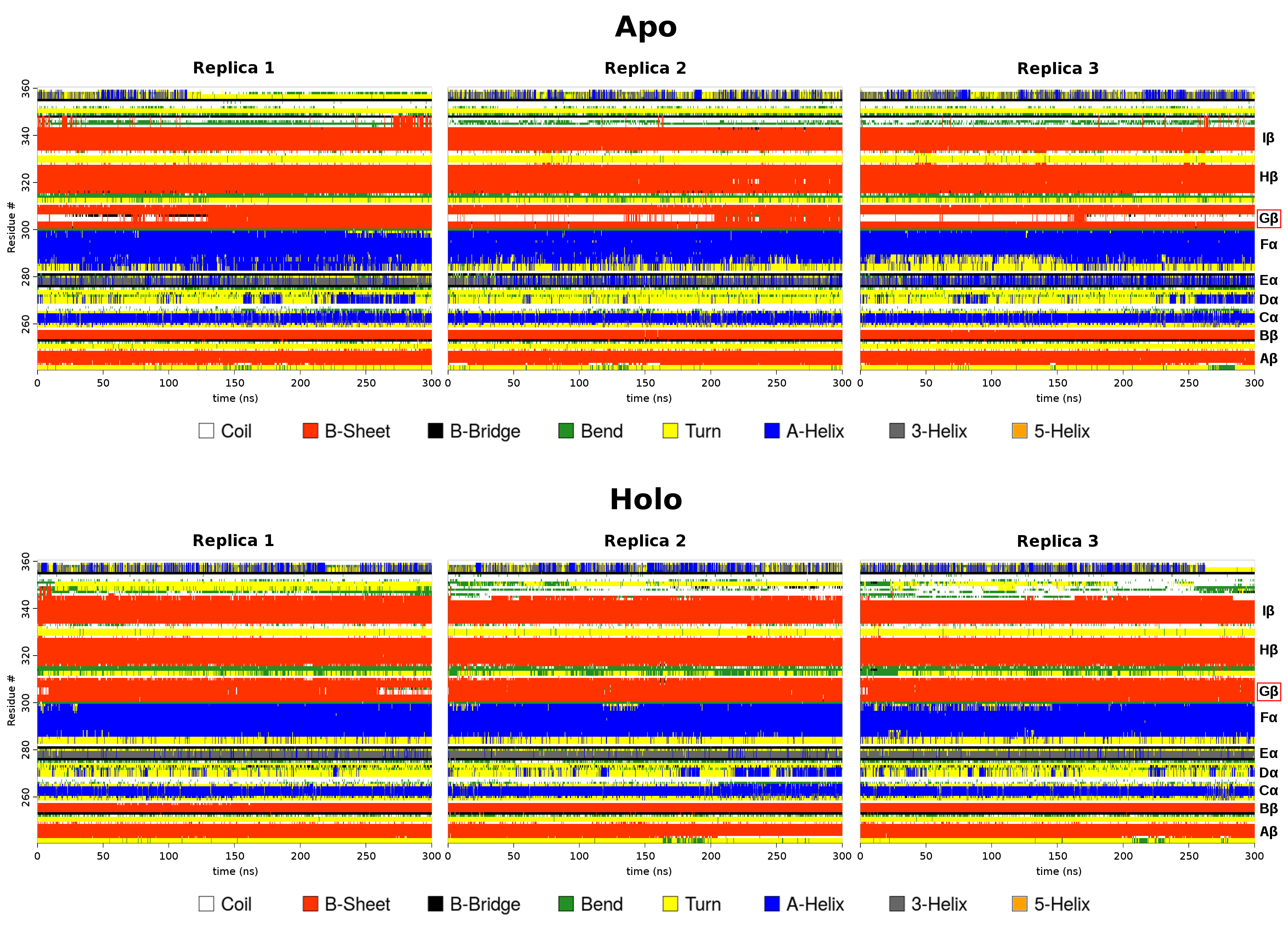

Supplement: S6 Fig — Secondary structure elements were assigned using the DSSP algorithm. The location of the Gβ element is indicated on the right-hand side. (TIF) [file pcbi.1006021.s006.tif]

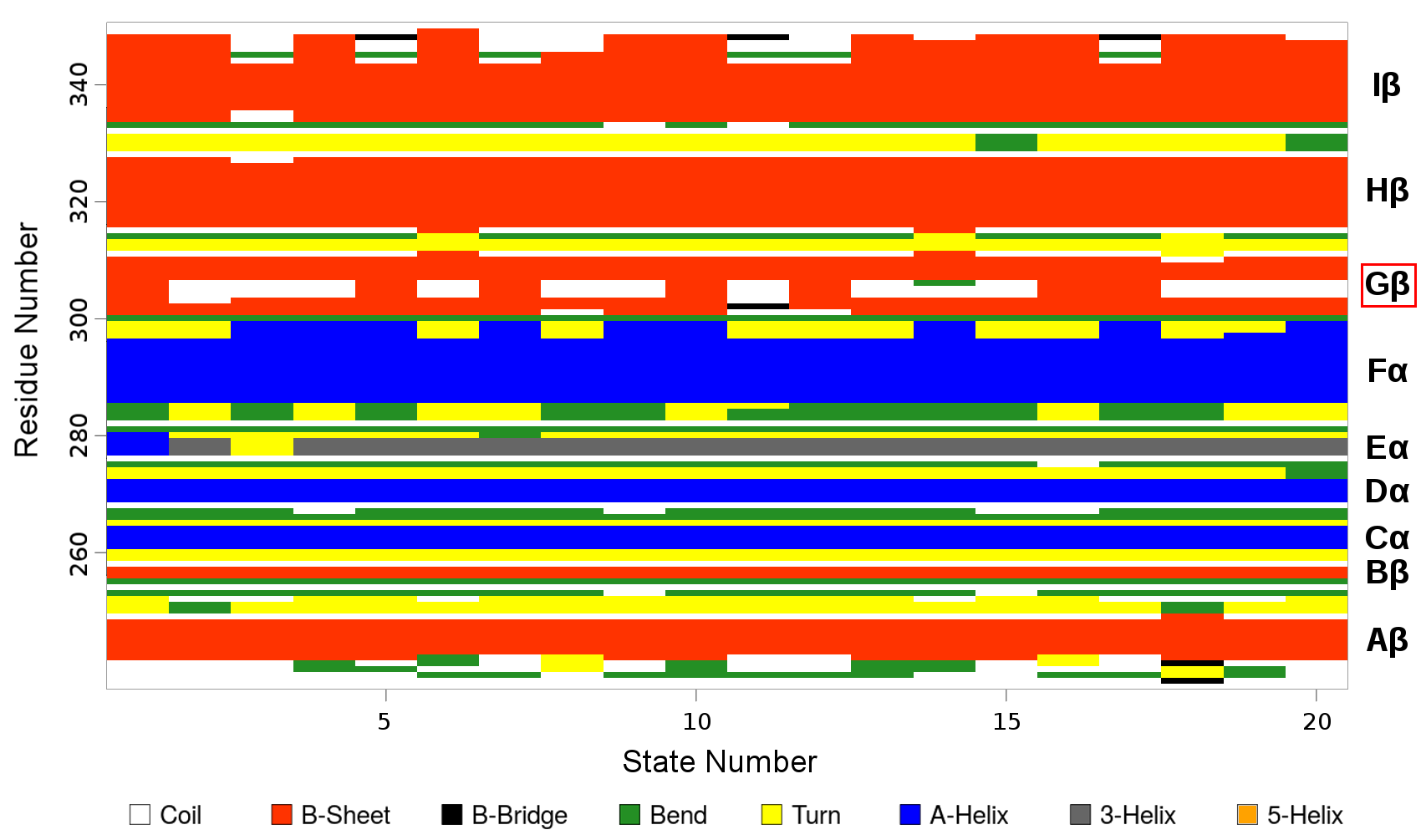

Supplement: S7 Fig — The secondary structure elements are labelled on the right according to the PAS domain nomenclature. (TIF) [file pcbi.1006021.s007.tif]

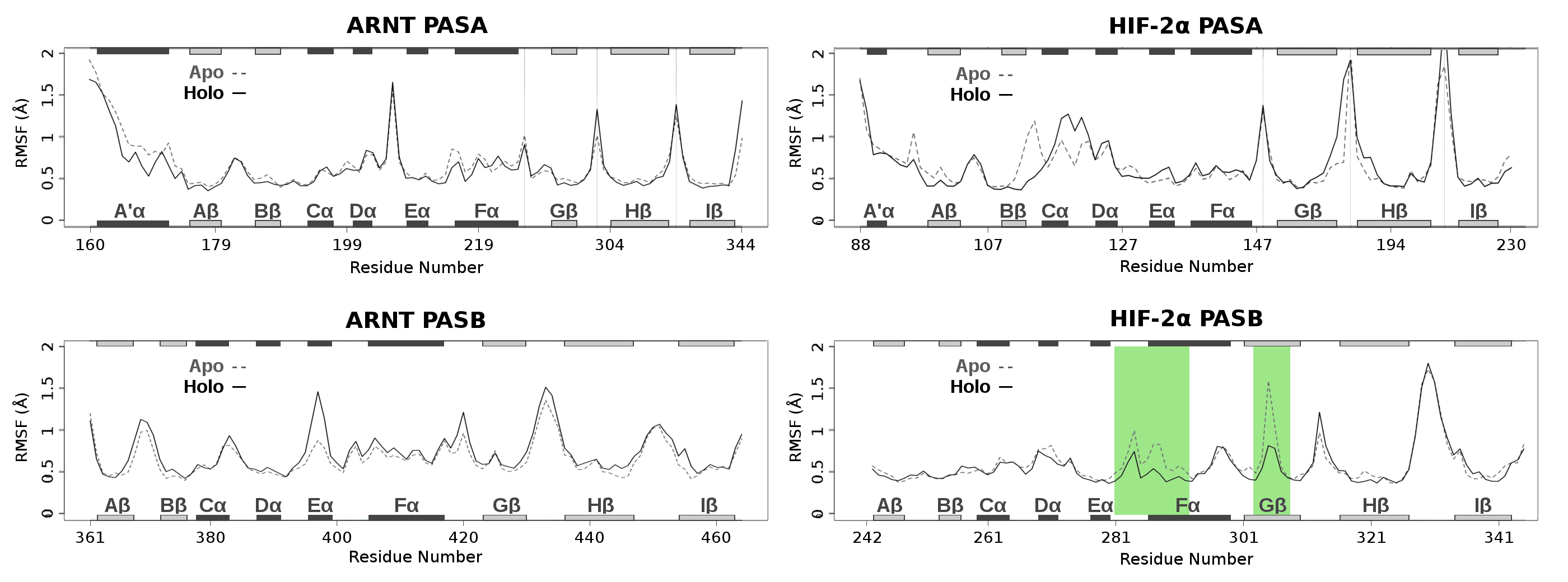

Supplement: S8 Fig — Ligand-perturbed regions discussed in the text are highlighted in light green. The long and highly flexible PAS-A loops are excluded from the calculation, and the corresponding gaps are indicated in the figure by vertical dashed lines. Secondary structure elements according to DSSP are reported on the top and bottom of the graphs (black: α-helix, light grey: β-strand). (TIF) [file pcbi.1006021.s008.tif]

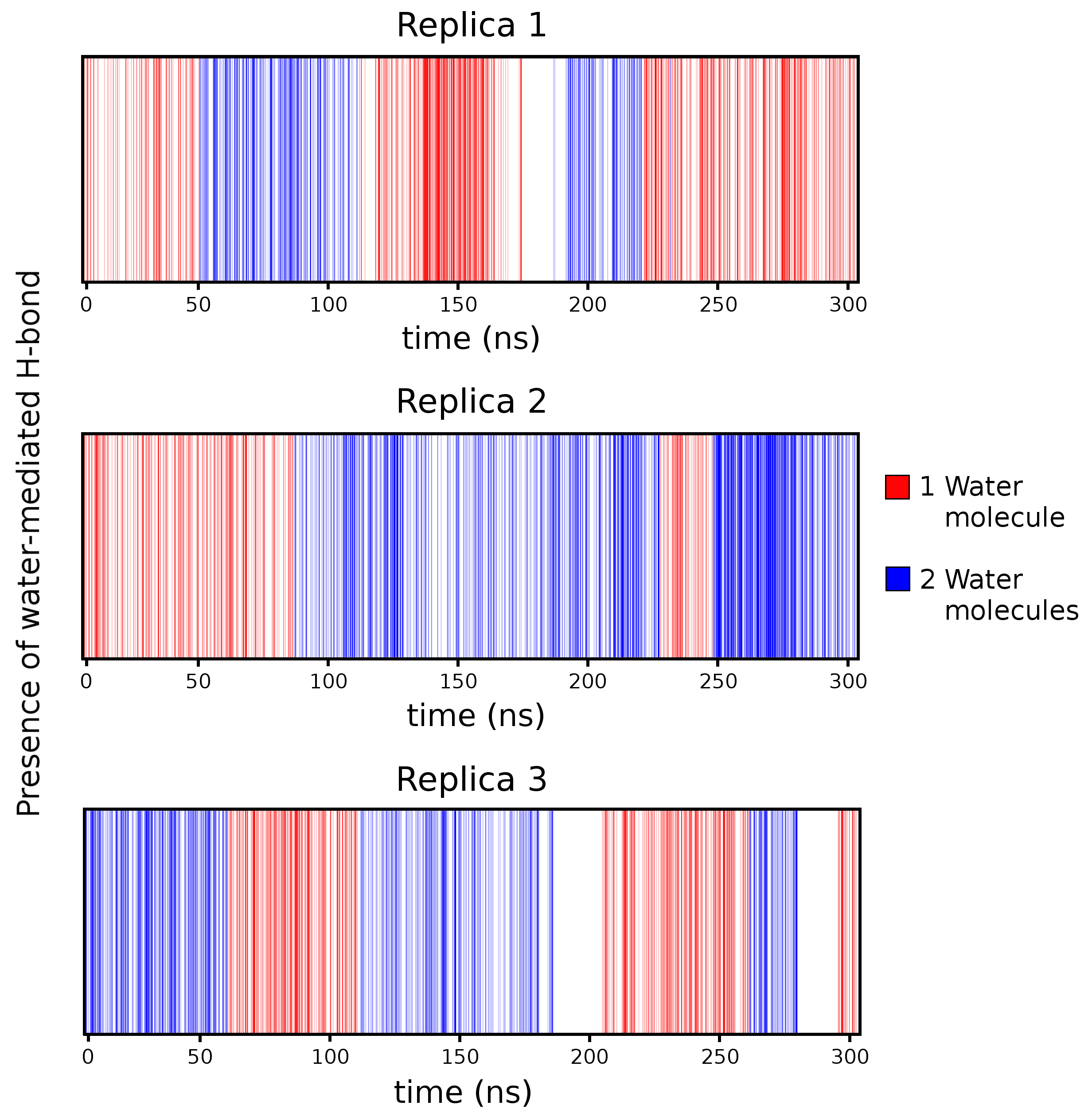

Supplement: S9 Fig — Interactions are present for the 36.4% of total simulation time. Red lines indicate an interaction mediated by one water molecule and blue lines indicate an interaction mediated by two water molecules. Geometric parameters for H-bond definition were chosen according to the GROMACS definition. (TIF) [file pcbi.1006021.s009.tif]

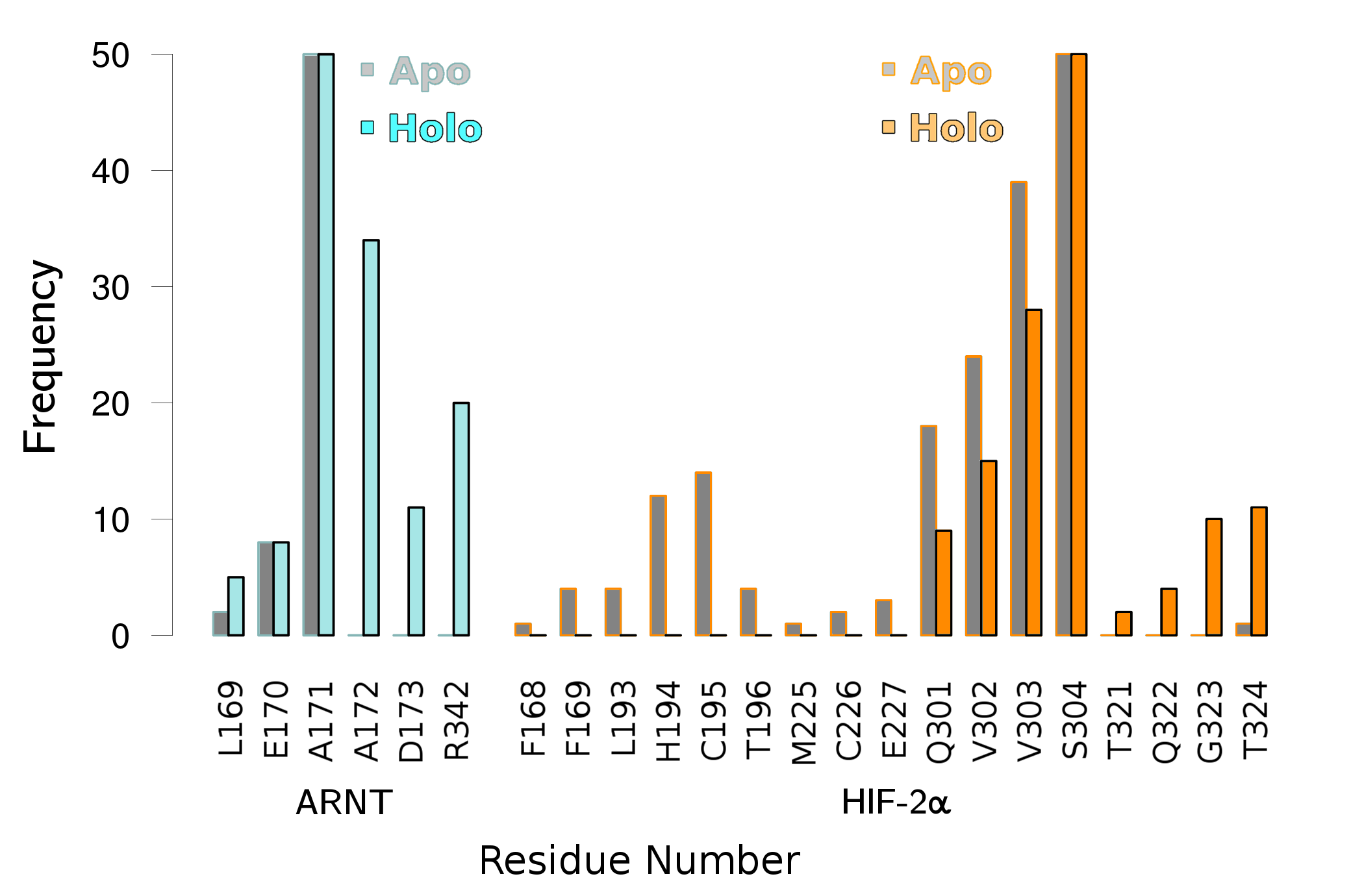

Supplement: S10 Fig — The frequency of each residue occurrence in the best 50 suboptimal paths is shown. (TIF) [file pcbi.1006021.s010.tif]

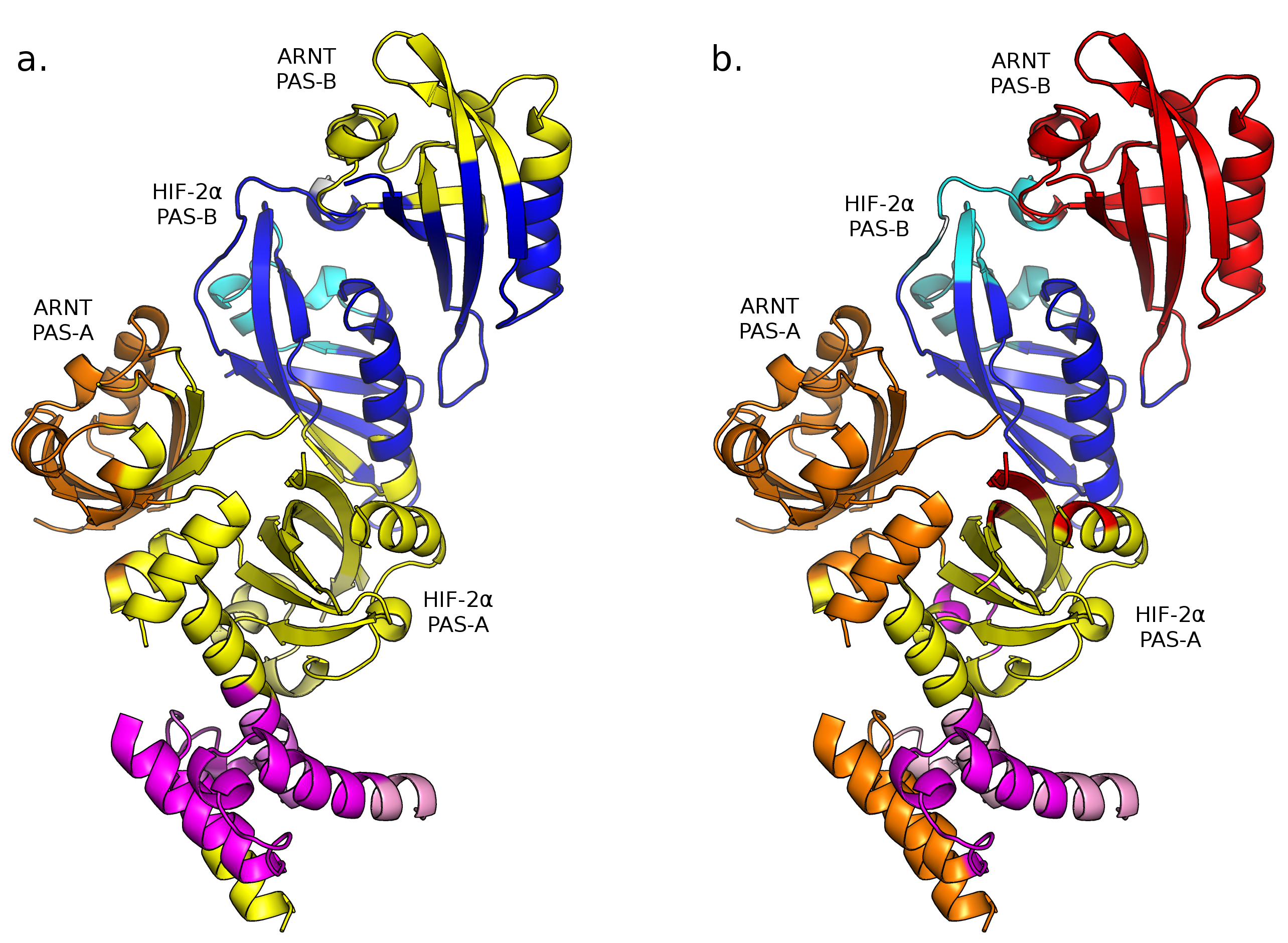

Supplement: S11 Fig — Residue positions are coloured according to the community membership. (TIF) [file pcbi.1006021.s011.tif]
