# Supplementary material for: Ligand-induced perturbation of the HIF-2α:ARNT dimer dynamics
Source: PLoS Comput Biol. 2018 Feb 28;14(2):e1006021. doi: 10.1371/journal.pcbi.1006021 (PMC5847239; doi:10.1371/journal.pcbi.1006021)
Supplement: S1 Table — (DOCX) [file pcbi.1006021.s012.docx]

# Supporting Information

**S1 Table**: UniRef codes of the amino acid sequences for ConSurf analysis.

| ARNT | |  | HIF-2α | |
| --- | --- | --- | --- | --- |
| Code | Residues |  | Code | Residues |
| A0A0P5T394 | 95-477 |  | UPI00075FFBE3 | 30-359 |
| UPI0004BDD6DC | 111-432 |  | UPI000643BBF5 | 26-360 |
| UPI0005B87606 | 98-416 |  | UPI000739CE23 | 29-358 |
| UPI0006827794 | 126-513 |  | L5LLB8 | 1-299 |
| UPI00065DC524 | 89-476 |  | A0A0S7KBH5 | 28-340 |
| I6QF65 | 63-425 |  | A0A0B5L0B1 | 26-359 |
| UPI000711CC77 | 98-463 |  | V9P247 | 28-355 |
| B7QNX4 | 32-381 |  | Q30HW1 | 28-356 |
| C7B7E8 | 16-380 |  | UPI00042BC0E8 | 1-303 |
| A0A139ZSV8 | 67-430 |  | UPI00046C2A70 | 28-356 |
| UPI0004BD6384 | 111-477 |  | UPI0006B31656 | 28-358 |
| A0A146KXM8 | 61-427 |  | UPI00064BBD62 | 40-374 |
| UPI00057691E6 | 79-445 |  | Q99814 | 26-360 |
| A0A067Y7W3 | 79-455 |  | A5H731 | 29-359 |
| UPI00064D0964 | 77-443 |  | D7R4L4 | 26-355 |
| UPI0005287B9C | 89-476 |  | UPI0004F00B20 | 26-357 |
| A0A0P5VLR8 | 60-426 |  | UPI000273AF39 | 347-682 |
| UPI0006428D62 | 89-454 |  | A0A146WXQ9 | 59-378 |
| UPI00065747EB | 118-484 |  | UPI0007425DF7 | 28-356 |
| G1T5P5 | 169-532 |  | S9WW67 | 22-385 |
| A0A131YRR4 | 82-440 |  | C0HAF0 | 76-405 |
| UPI000549A121 | 89-454 |  | A0A0S7KC54 | 28-356 |
| L5MDA5 | 33-365 |  | A5H735 | 28-358 |
| D3U1A8 | 81-446 |  | UPI0003C921D3 | 26-357 |
| A0A0S7JRS1 | 86-452 |  | W5UA58 | 28-359 |
| M4M6G6 | 63-426 |  | UPI00064E6330 | 110-444 |
| K1RFA8 | 96-458 |  | A0A1A8NTS3 | 28-353 |
| A0A075BJU4 | 88-451 |  | Q2PQU6 | 28-355 |
| UPI00062A7F4E | 74-440 |  | Q8QGM4 | 26-364 |
| UPI0006B35BAB | 85-451 |  | L5KEU0 | 18-347 |
| A0A0N8CTS1 | 60-426 |  | UPI0006D9332B | 32-360 |
| Q9W752 | 2-346 |  | UPI00064D36E1 | 77-411 |
| UPI000549A7D0 | 89-476 |  | A5H732 | 29-364 |
| UPI0005281C04 | 22-409 |  | T1Q068 | 28-359 |
| A0A0S7JRE1 | 86-452 |  | A0A0S7GPQ2 | 26-357 |
| UPI0003D0D839 | 98-464 |  | M9NZ78 | 28-358 |
| A0A0N8DW53 | 95-495 |  | UPI0004DFD5C7 | 25-352 |
| UPI000454375A | 324-690 |  | B3DJD1 | 26-357 |
| Q98SN3 | 98-485 |  | UPI0006520C79 | 26-357 |
| UPI0005225151 | 1-323 |  | V5IV35 | 26-348 |
| A0A0P5BRN5 | 22-356 |  | UPI0006B7017F | 32-362 |
| Q64FF9 | 72-439 |  | A0A0B5KUB3 | 26-359 |
| UPI0006D8E1D9 | 88-453 |  | A0A093LTT6 | 3-287 |
| UPI000739AFAE | 98-518 |  | UPI000328C85B | 26-360 |
| UPI00042C2352 | 92-458 |  | A0A1A8U738 | 26-360 |
| UPI000441D6C8 | 87-452 |  | A0A0B5KPE5 | 26-359 |
| UPI0005D237FF | 81-447 |  | UPI0006514FA8 | 33-367 |
| Q45FA8 | 72-439 |  | A5H729 | 28-359 |
| UPI0005CBD855 | 81-447 |  | UPI00062ABC7B | 26-356 |
| UPI00063F3AFD | 83-426 |  | A0A097NUX8 | 3-344 |
| UPI0001757CD6 | 86-453 |  | M4XYS7 | 26-359 |
| A0A146WSU9 | 124-490 |  | V5IV31 | 26-348 |
| A0A093CQS9 | 90-455 |  | T1Q067 | 28-358 |
| W8VLJ2 | 1-344 |  | UPI0005CC26CA | 61-391 |
| A0A147A6H2 | 80-469 |  | Q6EHI4 | 28-357 |
| Q1JPT1 | 72-439 |  | Q61221 | 29-358 |
| UPI0005762A31 | 79-445 |  | UPI0006B3D5E7 | 45-376 |
| A0A0P5SLH1 | 316-698 |  | UPI00071A8C6E | 26-360 |
| A0A0N8BLY7 | 170-552 |  | A5PJT1 | 26-360 |
| UPI00076A5B4F | 66-432 |  | A5H736 | 28-358 |
| W5JVY0 | 23-338 |  | A0MNY9 | 26-355 |
| UPI00074FFED5 | 108-473 |  | T1Q070 | 26-357 |
| M4WII3 | 43-413 |  | UPI000298DCA0 | 28-357 |
| A0A091TML3 | 60-425 |  | UPI000511B794 | 1-299 |
| A0A023FKI8 | 82-440 |  | Q6EMI3 | 14-342 |
| UPI0006B19A4D | 84-449 |  | T1Q049 | 28-358 |
| A0A0L0BUI2 | 22-386 |  | L7NCR4 | 28-357 |
| R9QBX3 | 61-422 |  | S5YDM3 | 29-359 |
| U5EX06 | 35-401 |  | UPI00074FECF5 | 31-360 |
| UPI0006B6D0D1 | 80-446 |  | G4WI71 | 28-356 |
| UPI00063C6E41 | 93-432 |  | T1Q050 | 26-357 |
| UPI0004EFC16D | 89-470 |  | V8NR02 | 32-339 |
| P27540 | 98-464 |  | UPI000661C0DF | 26-360 |
| A0A0S7JRT9 | 80-446 |  | G4WI69 | 26-357 |
| UPI000739C350 | 89-509 |  | D3XLB8 | 1-309 |
| A0A0P7UYA9 | 49-415 |  | UPI00074FF374 | 31-364 |
| B0X679 | 1-349 |  | Q6STN7 | 28-355 |
| Q2EJ28 | 74-432 |  | UPI0003F0D616 | 26-357 |
| A0A0P5VJQ8 | 95-477 |  | UPI000572B5AE | 45-376 |
| A0A0S7JRQ8 | 80-446 |  | A0A0Q3P931 | 68-397 |
| A0A0S7JTE2 | 26-392 |  | UPI0004D09713 | 26-360 |
| A0A0P6JSS4 | 125-524 |  | A0A0F7ZE51 | 26-359 |
| A0A146WTA3 | 266-632 |  | UPI000440629E | 39-373 |
| UPI0006B3A7CE | 85-451 |  | V9P1Z1 | 26-355 |
| A0A093D285 | 90-455 |  | UPI0004D04700 | 30-359 |
| P79832 | 74-440 |  | UPI0004ED0B1A | 26-357 |
| A0A087VEX9 | 60-425 |  | UPI00064401BE | 28-356 |
| A0A0P6DXW2 | 93-475 |  | Q2ESI0 | 26-365 |
| UPI00054B57F9 | 80-446 |  | A0A0P7YFP3 | 28-357 |
| A0A093PEA1 | 90-454 |  | B7SCS8 | 28-357 |
| A0A146WQN4 | 80-469 |  | UPI0003837EBD | 26-359 |
| A0A151M5M1 | 98-463 |  | UPI0007047B55 | 45-376 |
| UPI0006266D0F | 121-509 |  | A0A0P6K1K9 | 26-360 |
| UPI0005EE4D36 | 66-434 |  | UPI0004D05D0E | 26-357 |
| A0A0P5DP75 | 1-360 |  | F6M3M6 | 19-315 |
| Q3ULM2 | 83-449 |  | UPI000497AAB9 | 26-364 |
| A0A146RLJ8 | 80-446 |  | UPI000387DA3A | 70-401 |
| UPI000739D6FE | 89-487 |  | UPI000443C43F | 26-354 |
| UPI000522DF0F | 89-476 |  | UPI000643B575 | 26-354 |
| A0A0P5RAV2 | 118-484 |  | A0A093FZP8 | 1-287 |
| A0A0K2A1S8 | 98-464 |  | Q9Y2N7 | 26-357 |
| A0A146N347 | 80-446 |  | M7BBR3 | 1-309 |
| UPI000496B731 | 81-447 |  | T1Q069 | 27-355 |
| UPI0003833525 | 83-470 |  | UPI000642F46E | 26-354 |
| A0A0F8C586 | 96-410 |  | UPI0003F0B0D5 | 1-304 |
| A6NGV6 | 98-416 |  | D3XLB6 | 27-357 |
| A0A091Q7Y0 | 60-436 |  | UPI000649E977 | 62-349 |
| A0A146KT66 | 76-446 |  | UPI00048D8122 | 26-357 |
| M7BI69 | 92-420 |  | UPI000711F92A | 89-418 |
|  |  |  | A0A0B5L2C4 | 25-356 |
|  |  |  | UPI0004F4421F | 30-343 |
|  |  |  | A0A0S1LGL6 | 28-356 |
|  |  |  | T1Q053 | 26-354 |
|  |  |  | UPI0006549E8B | 26-357 |
|  |  |  | Q9YIB9 | 29-358 |
|  |  |  | Q3S3U2 | 29-359 |
|  |  |  | D7R4L3 | 28-349 |
|  |  |  | F1QNB9 | 27-354 |
|  |  |  | Q5IGQ2 | 1-326 |
|  |  |  | A1L531 | 27-361 |
|  |  |  | Q6PI33 | 28-357 |
|  |  |  | A0A151NWI5 | 26-359 |
|  |  |  | A0A091KDZ0 | 18-351 |
|  |  |  | B3FMV7 | 26-355 |
|  |  |  | UPI000494F56A | 28-359 |
|  |  |  | UPI0003EDFC47 | 29-358 |
|  |  |  | B4Y034 | 28-357 |
|  |  |  | A0A0A7RP54 | 14-318 |
|  |  |  | A0A091CYF4 | 64-393 |
|  |  |  | P97481 | 26-360 |
|  |  |  | A5H737 | 28-358 |
|  |  |  | UPI0006B72F41 | 26-356 |
|  |  |  | D7NII2 | 3-311 |
|  |  |  | V5IV44 | 26-348 |
|  |  |  | UPI00064CEB72 | 26-357 |
|  |  |  | UPI0004573AF8 | 26-356 |
|  |  |  | A0A146WXH3 | 28-356 |
|  |  |  | D6NF15 | 26-357 |
|  |  |  | Q0VBL6 | 24-355 |
|  |  |  | UPI000333AC72 | 54-388 |
|  |  |  | L9JA96 | 1-324 |
|  |  |  | Q5DJ37 | 28-355 |
|  |  |  | UPI00074FB983 | 32-366 |
|  |  |  | UPI00062ACAD7 | 26-356 |
|  |  |  | G5AUZ0 | 18-349 |
|  |  |  | UPI0006B32E3E | 99-433 |
|  |  |  | Q16665 | 29-358 |
|  |  |  | V9KF35 | 26-356 |
|  |  |  | UPI0005288C88 | 1-322 |
|  |  |  | UPI0004962FEC | 26-360 |
|  |  |  | V9KA26 | 34-363 |
|  |  |  | UPI0006D73943 | 59-390 |
|  |  |  | UPI0006441BB2 | 24-353 |
|  |  |  | UPI0004423AD8 | 32-361 |
|  |  |  | A0A067Y8N1 | 27-349 |
|  |  |  | D3XLB9 | 1-327 |
|  |  |  | L5KQ37 | 26-357 |
|  |  |  | A0A146N4E8 | 88-416 |
|  |  |  | UPI000642FFFD | 26-357 |
|  |  |  | V5IV55 | 26-348 |
|  |  |  | UPI000576227C | 26-358 |
|  |  |  | UPI00032AF43F | 26-357 |
|  |  |  | A0A1A7X1I2 | 28-353 |
|  |  |  | UPI00045421B6 | 41-370 |
|  |  |  | A0A088DJK3 | 29-360 |
|  |  |  | H9BIC8 | 1-303 |
|  |  |  | Q2PMW1 | 28-358 |
|  |  |  | Q98SW2 | 29-360 |
|  |  |  | A0A067Y838 | 27-349 |
