# Supplementary material for: Ligand-induced perturbation of the HIF-2α:ARNT dimer dynamics
Source: PLoS Comput Biol. 2018 Feb 28;14(2):e1006021. doi: 10.1371/journal.pcbi.1006021 (PMC5847239; doi:10.1371/journal.pcbi.1006021)
Supplement: S2 Table — (DOCX) [file pcbi.1006021.s013.docx]

# Supporting Information

**S2 Table:** Domain decomposition of the MM-GBSA ΔG_binding_ for the apo HIF-2α:ARNT dimer

|  | | | | ΔG_binding_ (kcal mol^-1^)  (SD)^a^ | Percent Values |
| --- | --- | --- | --- | --- | --- |
| Dimer | | | | -411.3 | - |
|  |  |  |  | (0,44) |  |
| ARNT | bHLH | | | -46.4 | 11.3 |
|  | Linker bHLH-PAS-A | | | -24.0 | 5.8 |
|  | PAS-A | Complete | -86.4 | | 21.0 |
|  |  | FG Loop | -26.0 | | 6.3 |
|  |  | GH Loop | -6.2 | | 1.5 |
|  |  | HI Loop | -2.4 | | 0.6 |
|  | Linker PAS-A-PAS-B | | | -16.7 | 4.1 |
|  | PAS-B | | | -34.5 | 8.4 |
| HIF-2α | bHLH | | | -47.8 | 11.6 |
|  | Linker bHLH-PAS-A | | | -13.5 | 3.3 |
|  | PAS-A | Complete | -60.7 | | 14.8 |
|  |  | FG Loop | -5.3 | | 1.3 |
|  |  | GH Loop | -1.2 | | 0.3 |
|  |  | HI Loop | -3.2 | | 0.8 |
|  | Linker PAS-A-PAS-B | | | -23.4 | 5.7 |
|  | PAS-B Complete | | | -57.4 | 14.0 |
|  | Linker C-term | | | -16.8 | 4.1 |

^a^ SD: standard error of the mean, defined as $\sigma/\sqrt{n}$, where n is the number of snapshots and $\sigma$ is the standard deviation between snapshots.
