# Supplementary material for: Ligand-induced perturbation of the HIF-2α:ARNT dimer dynamics
Source: PLoS Comput Biol. 2018 Feb 28;14(2):e1006021. doi: 10.1371/journal.pcbi.1006021 (PMC5847239; doi:10.1371/journal.pcbi.1006021)
Supplement: S3 Table — Contributions that differ by more than 0.5 kcal mol-1 are highlighted. (DOCX) [file pcbi.1006021.s014.docx]

# Supporting Information

**S3 Table:** Comparison of the per-residue decomposition of the MM-GBSA ΔG_binding_ between the apo and holo HIF-2α:ARNT dimers at the PAS-B:PAS-B interface. Contributions that differ by more than 0.5 kcal mol^-1^ are highlighted.

| ARNT | | |  | HIF-2α | | |
| --- | --- | --- | --- | --- | --- | --- |
| Residue | Apo | Holo |  | Residue | Apo | Holo |
|  | ΔG_binding_  (kcal mol^-1^) | |  |  | ΔG_binding_  (kcal mol^-1^) | |
| I364 | -1.18 | -0.82 |  | M250 | -1.99 | -2.00 |
| **R366** | **-1.40** | **-0.79** |  | **D251** | **-3.01** | **-2.28** |
| T374 | -2.13 | -2.08 |  | M252 | -0.55 | -0.67 |
| F375 | -6.57 | -6.19 |  | **K253** | **-1.34** | **-0.50** |
| D377 | 0.58 | 0.60 |  | S276 | -3.96 | -3.66 |
| H378 | -0.73 | -0.57 |  | **Y278** | **-4.91** | **-3.54** |
| **F446** | **-2.75** | **-1.77** |  | **E279** | **-1.65** | **-0.20** |
| Q447 | 0.53 | 0.43 |  | F280 | -0.52 | -0.32 |
| **N448** | **-2.14** | **-2.86** |  | Y281 | -2.58 | -2.86 |
| **P449** | **-4.45** | **-3.94** |  | **H282** | **-1.39** | **-0.78** |
| Y450 | -4.03 | -3.96 |  | A283 | -2.33 | -2.08 |
| E453 | 0.76 | 0.64 |  | L284 | -1.94 | -1.51 |
| **E455** | **-1.81** | **1.01** |  | **T290** | **-1.14** | **-0.58** |
| **Y456** | **-3.11** | **-1.29** |  | K291 | 0.69 | 0.77 |
| I458 | -0.61 | -0.28 |  | H293 | -2.42 | -2.33 |
|  |  |  |  | Q294 | -0.55 | -0.33 |
|  |  |  |  | C297 | -0.65 | -0.70 |
|  |  |  |  | L310 | -0.50 | -0.50 |
|  |  |  |  | W318 | -0.66 | -0.97 |
|  |  |  |  | E320 | 1.44 | 1.40 |
|  |  |  |  | L344 | -2.10 | -2.10 |
