# Supplementary material for: Ligand-induced perturbation of the HIF-2α:ARNT dimer dynamics
Source: PLoS Comput Biol. 2018 Feb 28;14(2):e1006021. doi: 10.1371/journal.pcbi.1006021 (PMC5847239; doi:10.1371/journal.pcbi.1006021)
Supplement: S4 Table — (DOCX) [file pcbi.1006021.s015.docx]

# Supporting Information

**S4 Table:** Mutations in the key regions for HIF-2α:ARNT dimerization and inhibition

|  | position | aa1 | aa2 | analysis | effect/prediction | ref |
| --- | --- | --- | --- | --- | --- | --- |
| ARNT PAS-A FG loop | | | | | | |
| ARNT | 238 | D | N | cancer-related genomic mutation | probably damaging | 70 |
|  | 253 | M | I | cancer-related genomic mutation | possibly damaging | 70 |
| HIF-2α | 247 | R | H | cancer-related genomic mutation | probably damaging | 70 |
|  | 258 | D | N | cancer-related genomic mutation | probably damaging | 70 |
|  | 338^a^ | M | E | ^15^N/^1^H HSQC spectra | reduces ARNT binding | 79,80 |
| HIF-2α PAS-B C-term | | | | | |  |
| HIF-2α | 355 | S | F | cancer-related genomic mutation | probably damaging | 70 |
|  | 359 | T | A | cancer-related genomic mutation | probably damaging | 70 |
| PAS-B:PAS-B interface | | | | | |  |
| ARNT | 366^b^ | R | A | co-IP | reduces dimer stability | 14,19 |
|  | 448^b^ | N | A | co-IP | reduces dimer stability | 14,19 |
|  | 452 | D | N | cancer-related genomic mutation | possibly damaging | 70 |
|  | 453 | E | K | cancer-related genomic mutation | possibly damaging | 70 |
|  | 456^b^ | Y | D | co-IP | reduces dimer stability | 14,19 |
|  |  |  | T | ^15^N/^1^H HSQC spectra | reduces HIF-2α binding | 81 |
| HIF-2α | 276 | S | L | cancer-related genomic mutation | probably damaging | 70 |
|  | 279 | E | V | cancer-related genomic mutation | probably damaging | 70 |
| HIF-2α PAS-B G-strand | | | | | |  |
| ARNT | 307 | H | Y | cancer-related genomic mutation | possibly damaging | 70 |
| HIF-2α | 304 | S | M | ITC K_D_ | reduces 0X3 binding | 24 |
|  | 306 | Q | L | cancer-related genomic mutation | probably damaging | 70 |
|  | 321 | T | I | cancer-related genomic mutation | probably damaging | 70 |
|  | 322^a^ | Q | E | ^15^N/^1^H HSQC spectra | reduces ARNT binding | 79,80 |
|  | 323 | G | E | cancer-related genomic mutation | probably damaging | 70 |
| HIF-2α PAS-B PAS-A:PAS-A communication path | | | | | | |
| ARNT | 167^b^ | L | E | co-IP | reduces dimer stability | 14,19 |
|  | 168^b^ | I | D | co-IP | reduces dimer stability | 14,19 |
|  | 171^b^ | A | D | co-IP | reduces dimer stability | 14,19 |
| HIF-2α | 169^c^ | F | D | co-IP | reduces dimer stability | 19 |
|  | 192^c^ | V | D | co-IP | reduces dimer stability | 19 |
|  | 194^c^ | H | A | co-IP | reduces dimer stability | 19 |
|  | 165 | E | K | cancer-related genomic mutation | possibly damaging | 70 |
|  | 223 | I | M | cancer-related genomic mutation | possibly damaging | 70 |

^a^ The effects of the triple mutant Q322E/M338E/Y342T of HIF-2α were analyzed by ^15^N/^1^H HSQC spectra (ref. 79); those of the corresponding triple mutant Q320E/V336E/Y340T of HIF-1α were evaluated both by ^15^N/^1^H HSQC (ref. 79) and co-IP experiments (ref. 80).

^b^ The dimer stability was evaluated for the following bHLH-PAS systems: HIF-2α:ARNT and HIF-1α:ARNT (ref. 19), NPAS1:ARNT and NPAS3:ARNT (ref.14)

^c^ The corresponding F168D, V191D and H193A mutants of HIF-1α were also evaluated by co-IP experiments.
